# Vibroacoustic Metamaterials for Low-Frequency Sound and Vibration Attenuation in Electric Vehicles: A Review

**DOI:** 10.3390/ma19153259

**Published:** 2026-08-01

**Authors:** Krisztian Horvath

**Affiliations:** Department of Vehicle Development, Audi Hungaria Faculty of Engineering, Széchenyi István University, Egyetem tér 1, 9026 Győr, Hungary; horvath.krisztian@ga.sze.hu

**Keywords:** vibroacoustic metamaterials, electric vehicles, NVH, locally resonant metamaterials, low-frequency vibration, lightweight structures

## Abstract

The transition from internal combustion engine vehicles to battery electric vehicles has changed the acoustic design problem in automotive engineering. The absence of combustion-related masking increases the perceptibility of tonal and narrowband sources, including gear whine, electric motor orders, inverter-related components, tire cavity resonances, auxiliary system noise, and lightweight-panel radiation. At the same time, mass-based acoustic treatments conflict with electric vehicle lightweighting, range, cost, and sustainability targets. Vibroacoustic metamaterials offer an alternative route by manipulating elastic and acoustic wave propagation through architected geometries, local resonances, periodicity, membranes, lattice architectures, and adaptive or topological wave-control mechanisms. This review examines vibroacoustic metamaterials for low-frequency electric vehicle noise, vibration, and harshness (EV NVH) from an engineering perspective. It covers mechanisms, EV-specific NVH problems, component applications, materials, manufacturing, modeling, validation, AI-assisted design, sustainability, and technology readiness. Particular emphasis is placed on order-targeted, path-oriented, manufacturable, and experimentally validated solutions for electric-drive (e-drive) housings, wheel arches, battery enclosures, body panels, covers, and auxiliary systems. The review concludes that vibroacoustic metamaterials are most promising when integrated into conventional NVH workflows through order analysis, transfer path ranking, robust resonator tuning, durability validation, and multi-objective design optimization.

## 1. Introduction

The transition from internal combustion engine vehicles to battery electric vehicles has fundamentally changed the acoustic design problem in automotive engineering. In conventional vehicles, combustion-related broadband noise often masked secondary mechanical, aerodynamic, and structural sources. In battery electric vehicles, this masking effect is largely absent, and the interior soundscape is therefore more exposed to tonal and narrowband components associated with the electric motor, inverter, reduction gearbox, tire–road interaction, auxiliary systems, and lightweight body structures [1,2,3,4]. This is a qualitative shift: tonal components become more noticeable, even when the overall level is lower. A recent thematic review of automotive NVH confirms the field-wide shift toward electrification, lightweight structures, multiphysics simulation, psychoacoustics, and data-driven development [5].

Throughout this review, low frequency denotes frequencies below 1000 Hz, with primary emphasis on the approximately 50–1000 Hz range relevant to vehicle structural vibration, tire cavity resonance, body booming, auxiliary system tones, and the lower orders of electric-drive excitation. This is an EV-NVH application-specific convention rather than a universal structural dynamics classification. Control in this range is especially difficult because acoustic and structural wavelengths are long, conventional porous treatments require impractical thicknesses, and the relatively low modal density of vehicle structures produces distinct resonances and local vibration patterns. In battery electric vehicles, the absence of combustion masking makes these tonal responses more perceptually prominent. Gear whine, motor-related electromagnetic orders, and tire cavity excitation are transferred through shafts, bearings, suspension interfaces, wheel arches, housings, and body structures into the cabin [6,7,8,9,10,11,12,13,14].

At the same time, lightweighting remains a central design objective in electric vehicle development. Lower structural mass contributes to improved energy efficiency, extended driving range, and potential reductions in battery size and resource consumption. However, thinner panels, optimized housings, lightweight battery enclosures, and composite structures may exhibit reduced damping, altered modal behavior, and increased acoustic radiation efficiency. Conventional NVH countermeasures such as mass loading, thick barriers, constrained-layer damping, and large tuned absorbers can remain effective in selected applications, but they often conflict with weight, packaging, temperature, cost, and manufacturability constraints [6,7,8,9,13,14]. Metamaterials should therefore be viewed as a targeted complement rather than a universal replacement: conventional absorbers and damping treatments remain preferable for many broadband problems, whereas metamaterial concepts are most attractive when a narrow low-frequency band must be controlled under strict mass and space constraints. The recent vehicle sound-package literature likewise shows that absorbers, barriers, damping layers, seals, and multilayer systems remain essential reference solutions rather than obsolete technologies [15].

Vibroacoustic metamaterials offer a different route to noise and vibration attenuation. Instead of relying primarily on added mass or intrinsic damping, these materials manipulate elastic and acoustic wave propagation through engineered geometry. Periodic unit cells, local resonators, membrane-mass systems, auxetic lattices, and topological architectures can create frequency regions in which waves are strongly attenuated or prevented from propagating. These regions are commonly described as bandgaps or stop bands. The theoretical basis of this behavior originates in the study of wave propagation in periodic structures, phononic crystals, and locally resonant sonic materials [16,17,18,19,20,21,22,23,24,25,26,27,28,29].

For electric vehicle applications, locally resonant metamaterials are especially important because they can operate in a subwavelength regime. Pure Bragg-type phononic crystals generally require lattice dimensions comparable to the wavelength of the target wave, which is impractical for many low-frequency vehicle problems. By contrast, locally resonant systems use internal oscillators that interact dynamically with the host structure near their resonance frequency. This enables compact solutions for frequencies that would otherwise require large absorbers or substantial mass addition [18,19,20,21,22,23,24,25,26,27].

Recent automotive studies suggest that this concept can be translated from idealized beams and plates toward vehicle-relevant components. Reported examples include metal 3D-printed resonant elements for wheel-arch tire-noise mitigation, locally resonant lightweight gearbox housings, acoustic metamaterial treatments for body panels, vibroacoustic metamaterials for vehicle doors, power-electronics covers, and lattice or auxetic structures for battery pack vibration mitigation [6,7,8,9,30,31,32,33,34,35]. These studies do not yet constitute a mature production technology across all vehicle subsystems, but they demonstrate that metamaterial-based NVH solutions can be aligned with realistic automotive source–path–receiver problems.

The main challenge is robustness. Vibroacoustic metamaterials are sensitive to geometry, damping, resonator tuning, boundary conditions, attachment stiffness, manufacturing scatter, temperature, humidity, fatigue loading, and aging. In a laboratory unit cell, the attenuation mechanism may be clear, but the same concept can behave differently when integrated into a curved, bolted, damped, thermally loaded, and geometrically non-uniform vehicle component. Automotive use therefore requires more than bandgap prediction. It also needs component simulation, transfer path analysis, durability testing, and manufacturable design [6,7,8,9,29,30,31,32,33,34,35,36,37,38,39,40,41,42,43,44,45,46].

Artificial intelligence and data-driven design methods are becoming increasingly relevant because the design space of vibroacoustic metamaterials is large and strongly nonlinear. Surrogate models, genetic algorithms, topology optimization, deep learning inverse design, and digital twin-inspired workflows can reduce the number of expensive finite element (FE) simulations and support order-targeted tuning of resonators to measured or simulated EV excitation content [36,37,38,47,48,49,50,51,52,53]. For EVs, known motor-, gear mesh-, and tire-related orders can be mapped to target attenuation bands over the relevant operating-speed range into target attenuation bands.

Existing reviews have primarily emphasized general acoustic metamaterial physics, phononic crystals, local-resonance mechanisms, or broad classes of dynamic mechanical metamaterials [26,27,28,29,52]. They provide essential foundations, but they generally give less attention to the complete EV engineering chain linking order-related sources, structural transfer paths, component integration, scalable manufacturing, environmental durability, and vehicle-level validation. The present review addresses this gap by evaluating metamaterial concepts through the constraints and decision criteria used in automotive NVH development. A recent transportation-focused review similarly identifies scale-up, lightweight integration, and realistic validation as central barriers to practical acoustic metamaterial deployment [54].

Accordingly, the objectives are to: identify the metamaterial mechanisms most suitable for EV-relevant frequencies below 1000 Hz; critically compare their advantages, limitations, and component applicability; synthesize the available quantitative automotive evidence; evaluate manufacturing, durability, sustainability, and technology-readiness constraints; and clarify how unit-cell and component models can be integrated with order analysis, transfer path analysis, and vehicle-level validation. The distinctive contribution is therefore an EV-specific engineering decision framework rather than another mechanism-only catalogue.

Beyond the scope of existing mechanism-focused reviews, the present article contributes five EV-specific elements: (i) an application-specific definition of low-frequency EV NVH and a source–path–receiver framing of the problem; (ii) mechanism-to-application and engineering-selection matrices for vehicle components; (iii) a quantitative synthesis of reported automotive attenuation evidence and validation maturity; (iv) an integrated assessment of manufacturing, durability, sustainability, and technology readiness; and (v) an order-targeted development logic linking excitation identification, transfer path ranking, metamaterial design, component testing, and vehicle-level validation.

## 2. Review Scope and Methodology

The paper follows a structured, engineering-oriented narrative review approach with explicit search, screening, and data-extraction steps. Its scope is broader than a conventional acoustic material survey because it considers vibroacoustic metamaterials as multifunctional vehicle components rather than as isolated laboratory specimens. The literature covers acoustic and elastic metamaterials, phononic crystals, architected materials, automotive NVH, additive and conventional manufacturing, validation, optimization, and machine learning (ML).

The review is organized around five questions. First, which physical mechanisms are most relevant for low-frequency sound and vibration attenuation in electric vehicles? Second, which vehicle components have already been investigated using metamaterial concepts? Third, which materials and manufacturing routes are realistic for automotive deployment? Fourth, which numerical and experimental methods are required to connect unit-cell bandgaps to vehicle-level NVH performance? Fifth, how can AI-assisted design methods accelerate the development of order-targeted, lightweight, and manufacturable meta-structures?

### Search Strategy and Study Selection

The formal literature search was completed on 10 July 2026 in the Web of Science Core Collection, Scopus, SAE Mobilus, and IEEE Xplore. Google Scholar was used only for supplementary citation checking and was not included in the formal record count. The search covered 2000–2026, where supported by the database interface; the Scopus search was restricted to 2006–2026. The principal Boolean expression combined three blocks: (“vibroacoustic metamaterial*” OR “acoustic metamaterial*” OR “elastic metamaterial*” OR “locally resonant metamaterial*” OR “phononic crystal*” OR metastructure*) AND (“electric vehicle*” OR automotive OR vehicle OR gearbox OR “e-drive” OR “battery enclosure” OR “wheel arch” OR tyre OR tire) AND (NVH OR vibration OR noise OR acoustic* OR “sound radiation” OR “structure-borne”). Database-specific field codes were adapted without changing the conceptual search.

The four formal database searches identified 410 records: 139 in Web of Science, 244 in Scopus, 19 in SAE Mobilus, and 8 in IEEE Xplore. Records were exported in RIS or BibTeX format and deduplicated using DOI and normalized bibliographic fields. Removal of 132 duplicate records left 278 unique records for title-and-abstract screening. Records meeting the scope criteria were then assessed for full-text relevance. The final synthesis retained 101 cited sources, including database-screened journal and conference papers together with foundational books, standards, and technical sources identified through backward and forward citation checking. Studies were eligible when they provided direct automotive or EV relevance, or a transferable low-frequency acoustic or elastic metamaterial mechanism with a clear vehicle application. Purely optical, electromagnetic, or thermal metamaterial studies without an acoustic or elastic-wave-control contribution, duplicate records, non-scholarly promotional material, and records lacking sufficient technical detail were excluded.

The literature base was compiled from foundational publications on acoustic and elastic metamaterials, review papers on phononic and locally resonant systems, automotive NVH studies, component-level vehicle demonstrators, manufacturing-oriented studies, standards relevant to acoustic and vibration testing, and recent work on machine learning-based inverse design. This combination was selected to avoid a purely generic metamaterial overview and to keep the discussion centered on electric vehicle NVH implementation.

Studies were prioritized when they met at least one of the following criteria: direct relevance to electric vehicle or automotive NVH; demonstration of locally resonant, phononic, membrane-type, auxetic, topological, or lattice-based attenuation mechanisms; relevance to low-frequency or low-to-mid-frequency vibration; use of manufacturable or automotive-compatible materials; inclusion of experimental validation; or use of data-driven, topology-optimized, or inverse-design methods. Foundational studies were retained when they established mechanisms required for interpreting later automotive work. Greater evidentiary weight was assigned to studies reporting component- or vehicle-level validation, quantitative attenuation, mass information, manufacturing details, and environmental or durability testing.

The discussion separates three maturity levels. Fundamental studies establish physical wave-control mechanisms but may use idealized periodic media, beams, plates, or impedance-tube samples. Component-level studies apply the concept to a realistic vehicle subsystem such as a gearbox housing, wheel arch, door, cover, or battery casing. Vehicle-level studies evaluate the effect within a broader source–path–receiver environment. This distinction is important because a strong unit-cell bandgap does not automatically imply a measurable reduction in cabin sound pressure level.

The review also treats manufacturing and durability as core scientific criteria rather than as afterthoughts. Automotive metamaterials must survive thermal cycling, humidity, salt spray, road debris, vibration fatigue, dimensional variation, adhesive aging, and possible corrosion. Therefore, the evaluation of each concept includes not only attenuation performance but also the likely production route, material stability, integration constraints, quality-control difficulty, and lifetime robustness [39,40,41,42,43,44,45,46].

AI is treated as a design aid, not as a separate topic. ML, surrogate modeling, generative design, and topology optimization are evaluated according to their ability to shorten the development loop, connect target frequencies to real EV order content, and include manufacturing or durability constraints. In this sense, AI is most useful when it supports a physics-informed NVH workflow, rather than acting as a black-box geometry generator [36,37,38,47,48,49,50,51,52,53].

## 3. Theoretical Foundations of Vibroacoustic Metamaterials

Vibroacoustic metamaterials (VAMMs) are architected systems whose dynamic response depends on both constituent properties and a deliberately designed geometry. Periodicity, local resonators, membranes, cavities, lattices, and multi-material layouts provide additional control over elastic and acoustic wave propagation beyond that available in homogeneous materials [26,27,28,29].

For low-frequency EV NVH, the central passive mechanisms are Bragg scattering and local resonance. Bragg attenuation requires wavelength-scale periodicity, whereas compact resonators can create subwavelength stop bands. Their practical distinction is developed in Section 3.2 and Section 3.3, while the figures retained in this review prioritize quantitative synthesis and representative published automotive evidence.

The most important theoretical concept for the present review is the frequency bandgap, also referred to as a stop band. Within such a band, wave propagation through the structure is strongly attenuated because of destructive interference, localized dynamic effects, negative effective properties, or topologically induced wave confinement. For electric vehicle NVH, the usefulness of a bandgap depends less on the existence of an ideal mathematical stop band and more on whether the attenuation overlaps with a relevant order, tonal component, structural mode, or transfer path in the vehicle [1,6,7,8].

### 3.1. From Conventional Materials to Architected Wave-Control Systems

Unlike porous absorbers and viscoelastic layers, which rely mainly on intrinsic dissipation, VAMMs use geometry to tailor wave transmission. This enables tuning to gear mesh harmonics, tire cavity resonances, motor orders, or panel modes, but it also makes performance sensitive to geometric scatter, attachment stiffness, polymer modulus, and damping [6,27,29,38,55].

The theoretical foundation of vibroacoustic metamaterials therefore needs to be read together with practical constraints. An ideal unit-cell bandgap is not sufficient for automotive deployment. The design must also maintain structural stiffness, survive fatigue loading, tolerate temperature and humidity variation, and remain manufacturable in a repeatable production process [8,33,46].

### 3.2. Phononic Crystals and Bragg Scattering

Phononic crystals are periodic elastic or acoustic media in which spatially repeated impedance contrasts create frequency bands where waves cannot propagate. The mechanism is analogous to wave filtering in periodic electrical or optical systems and has roots in classical wave propagation theory for periodic structures [16,17]. In elastic media, the existence of acoustic and elastic band structures was demonstrated by early studies on periodic composites and later became a central theme of phononic-crystal research [18,19].

In a simplified one-dimensional interpretation, the Bragg bandgap center is approximately $f_{Bragg}\approx \frac{c}{2a}$, where *c* is wave speed and *a* is lattice spacing. The relation explains why compact periodicity is feasible at high frequencies but becomes impractically large at low frequencies [29,56].

Below approximately 1000 Hz, the required Bragg periodicity can exceed the available space in housings, wheel arches, panels, and battery enclosures. Pure Bragg designs are therefore mainly relevant at higher frequencies or in hybrid and lattice systems that combine periodicity with local resonance [29,56,57,58,59].

### 3.3. Locally Resonant Metamaterials

Locally resonant metamaterials were introduced to overcome the wavelength-scale limitation of Bragg scattering. Instead of relying primarily on destructive interference from periodic impedance contrasts, they use internal resonators that interact dynamically with the host structure. The landmark locally resonant sonic material of Liu et al. demonstrated that a bandgap can be produced at wavelengths much larger than the lattice spacing, which is the central physical reason for the relevance of this class to compact low-frequency applications [20]. Subsequent theoretical and experimental work clarified the role of negative effective density, negative effective modulus, and resonant inclusions in wave attenuation [21,22,23,24,25,26].

The simplest engineering model is a mass-spring resonator attached to or embedded in a host structure. Its resonance frequency is $f_{0}=\frac{1}{2\pi }\sqrt{\frac{k}{m}}$, where *k* is the effective stiffness and *m* is the resonator mass. Near resonance, the resonator can oscillate out of phase with the host motion. This produces a reactive force that reduces wave transmission and may create a stop band when repeated across a structure. In this sense, a locally resonant metamaterial can be interpreted as a distributed and integrated tuned vibration absorber [20,22,26].

For automotive NVH, distributed resonators can avoid the large concentrated masses of low-frequency tuned mass dampers and create a designed attenuation band across a panel, casing, cover, or wheel-arch region. Their main weakness is detuning: mass, stiffness, damping, attachment quality, boundary conditions, temperature, aging, and manufacturing scatter can shift or weaken the stop band [6,7,38,44,45,46].

### 3.4. Membrane-Type Acoustic Metamaterials

Membrane-type acoustic metamaterials combine a tensioned membrane with attached or embedded masses. Membrane tension and local inertia can create high low-frequency transmission loss or reflection in a thin structure, making the concept attractive for lightweight trim, liners, and covers where structural load bearing is secondary [23,26]. Recent systems combine low-frequency absorption and vibration isolation, membrane and local-resonance mechanisms, transparent multifunctional panels, and configurable resonant barriers, illustrating the breadth of thin or multifunctional concepts available beyond a single membrane-mass cell [60,61,62,63].

Nevertheless, membrane-type systems require careful automotive interpretation. Pre-tension, polymer stiffness, edge clamping, humidity, thermal aging, and fatigue can significantly affect resonance frequency. A membrane that performs well under laboratory boundary conditions may not retain the same response after repeated thermal cycling or long-term vehicle exposure. Therefore, membrane-type solutions are promising but need stronger durability and environmental validation before they can be treated as production-ready EV NVH components [46].

### 3.5. Helmholtz, Labyrinthine, and Coiled-Space Acoustic Concepts

Helmholtz resonators, labyrinthine channels, and coiled-space concepts use compact cavity resonance or extended acoustic paths to interact with frequencies that would otherwise require much thicker packages. In EVs, they are most relevant to covers, HVAC components, power-electronics enclosures, and secondary acoustic packages [14,26,27,28].

A practical research direction is the integration of Helmholtz-type cavities with locally resonant structural features. In such hybrid designs, the acoustic cavity can address airborne transmission while the structural resonator controls bending waves. This dual-path approach is particularly relevant for EV components that radiate both structure-borne and airborne tonal noise.

### 3.6. Lattice, Auxetic, and Pentamode Mechanical Metamaterials

Lattice-based mechanical metamaterials obtain their effective properties from cellular architecture. By changing strut orientation, cell size, relative density, nodal geometry, and material distribution, designers can tune stiffness, mass distribution, energy absorption, and wave propagation behavior. Modern lattice metamaterials are strongly connected to additive manufacturing because many high-performance architectures are difficult to fabricate using conventional subtractive processes [64,65,66,67,68].

Auxetic materials are a particularly important subclass because they exhibit a negative Poisson ratio. Instead of contracting laterally under tensile loading, they expand. Lakes demonstrated negative-Poisson ratio foam structures, and subsequent work clarified the broader relevance of Poisson ratio control in modern materials [69,70]. For vehicle components, auxetic architectures are attractive because they may combine vibration attenuation, indentation resistance, crash-energy absorption, and lightweight design [55,67]. Recent reviews of biomimetic auxetic systems further emphasize the need to evaluate vibration attenuation together with structural load capacity, deformation mode, and manufacturability [71].

Pentamode materials represent another branch of mechanical metamaterials with unusual effective elastic behavior. Their potential lies in the possibility of tailoring bulk and shear responses almost independently. For vibroacoustic applications, this can support impedance manipulation, vibration isolation, and structural–acoustic control, although practical deployment requires careful consideration of strength, fatigue, and manufacturability [39,72,73].

### 3.7. Hybrid and Multi-Resonant Meta-Structures

Hybrid and multi-resonant systems use resonators with different masses, stiffnesses, beam lengths, or cavity volumes so that adjacent attenuation bands cover several tones or a limited frequency sweep. This is useful for speed- and load-dependent EV sources, although the added elements increase mass and tuning complexity [6,7,33]. Self-tuning mass architectures and comparative lattice-geometry studies illustrate two complementary routes for increasing robustness or widening the useful low-frequency range [74,75].

Hybridization can also combine structural and acoustic mechanisms. For example, a sandwich structure may include locally resonant beams for bending-wave control and acoustic cavities for airborne transmission loss. Similarly, acoustic black hole concepts can be combined with local resonators to concentrate and dissipate bending-wave energy. These approaches offer broader performance but increase design complexity, manufacturing requirements, and the risk of unwanted coupling or local amplification outside the target band [26,33].

### 3.8. Topological Acoustic and Elastic Metamaterials

Topological acoustic and elastic metamaterials use band-structure topology to guide waves along selected interfaces with relative robustness to disorder, bends, and defects. Their automotive use remains exploratory, but the concept may eventually support defect-tolerant wave paths that redirect vibration away from efficient radiating regions [27,29,59].

Potential applications include waveguiding strips in floor structures, protected vibration paths around battery enclosures, and defect-tolerant acoustic liners. The difficulty is that topological behavior usually requires carefully designed periodicity, symmetry control, and interface definition. These requirements must be reconciled with curved panels, stamped structures, joints, holes, local reinforcements, and manufacturing tolerances in vehicle bodies.

### 3.9. Active, Adaptive, and Tunable Metamaterials

Active, adaptive, and tunable metamaterials vary effective stiffness, damping, electrical impedance, or resonance frequency to address speed-dependent motor, inverter, and gear orders. Candidate approaches include piezoelectric shunts, adjustable resonators, magnetic elements, shape-memory systems, and active feedback [36,37,49].

The advantage of active or tunable systems is their ability to follow moving orders. The disadvantage is system complexity. Sensors, control electronics, power supply, fail-safe behavior, thermal robustness, electromagnetic compatibility, diagnostic requirements, and cost must be addressed before such concepts can be adopted in production vehicles. Therefore, active metamaterials are best considered as a longer-term solution for high-value tonal problems rather than as immediate replacements for passive NVH treatments.

### 3.10. Mechanism Selection for Electric Vehicle NVH Applications

No metamaterial family is universally superior. Local resonance is the most direct option for compact fixed-frequency attenuation; hybrid systems address several nearby tones; membrane and cavity concepts suit thin covers and acoustic packages; lattice, auxetic, and pentamode structures are strongest when NVH is combined with structural functions; and topological or tunable systems offer lower-maturity capabilities. Selection should begin from the source–path–receiver problem and the stability of the target frequency [6,7,8,23,26,27,34,36,37,49] (Table 1).

**Figure 1:**
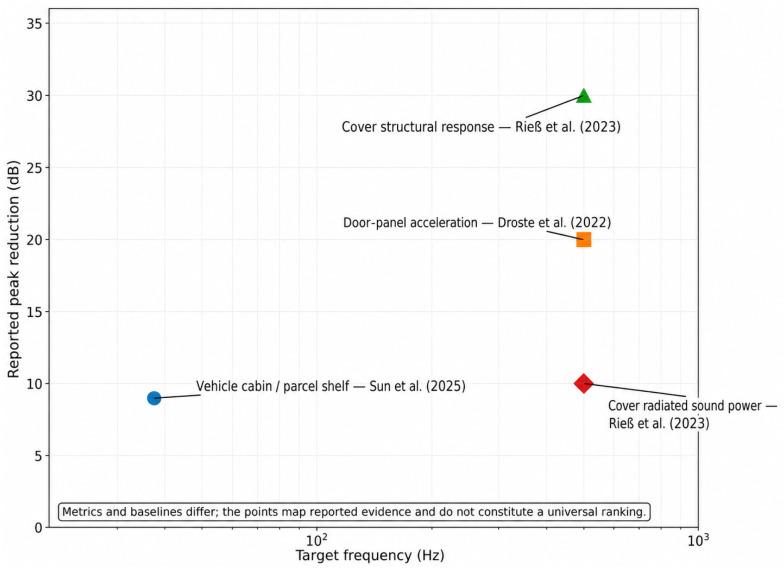
Literature-derived quantitative evidence for representative automotive vibroacoustic metamaterial studies. The points show reported peak reductions for a vehicle parcel-shelf/body-boom treatment, vehicle-door demonstrators, and an electric-powertrain electronics cover [30,31,33]. Marker shapes distinguish vehicle response, component acceleration, structural vibration, and radiated sound-power metrics. Because the baseline structures, excitation conditions, and measured quantities differ, the figure should be interpreted as an evidence map rather than a universal performance ranking.

Electric vehicles require a frequency-targeted interpretation of NVH because dominant sources are often tonal, order-related, and weakly masked. To complement the mechanism comparison with published application evidence, Figure 1 maps the reported peak reductions from representative automotive studies for which both a target frequency and a reduction in decibels could be extracted. The plotted results use different response quantities and reference conditions; accordingly, they identify the scale and location of reported effects rather than provide a direct ranking of the technologies [30,31,33].

## 4. Low-Frequency NVH Challenges in Electric Vehicles

Frequencies below approximately 1000 Hz are difficult to control in EVs because wavelengths are long, structural modes are sparse, porous absorbers are inefficient at practical thicknesses, and tonal components are weakly masked. The relevant phenomena include road-induced body vibration, tire cavity resonance, low-order drivetrain and auxiliary tones, panel booming, and the lower motor- and gear-order range; psychoacoustic quality therefore depends on tonal prominence as well as overall dB(A) [1,2,3,4,6,13,14,40,41].

This chapter organizes the most important low-frequency EV NVH challenges from a source–path–receiver perspective. The objective is not to replace conventional NVH terminology, but to translate the vehicle-level problem into metamaterial design requirements: target frequency, spatial location, dominant wave type, attachment condition, expected temperature range, and acceptable mass or packaging penalty.

### 4.1. Electric Motor Orders and Electromagnetic Force Excitation

Electric traction motors generate periodic electromagnetic forces associated with slotting, pole-pair interactions, magnetic field harmonics, torque ripple, and inverter excitation. These forces act on the stator and rotor and can excite the motor frame, bearing supports, e-drive housing, and neighboring structural components. Because these excitations are order-related, their frequencies change with rotational speed and may sweep through structural resonances during acceleration or deceleration [1,6,13].

A fixed passive stop band is suitable only when a critical operating range is narrow. Wider order sweeps favor multi-resonant, graded, or tunable solutions, usually placed in accessible housing walls, covers, brackets, or transfer path components rather than inside the electromagnetic machine [6,26,27,29,31,36,52]. A recent EV-focused split-ring-resonator study demonstrates a complete-bandgap concept intended to address airborne and multiple structure-borne wave polarizations associated with inverter-related tonal excitation [76].

### 4.2. Inverter and Power-Electronics Tonal Noise

Power electronics can contribute to EV tonal noise through inverter switching, electromagnetic interaction with the motor, and vibration of covers or mounting structures. Although switching frequencies may lie above the low-frequency range, their sidebands, structural coupling, and cover-panel resonances can create audible tonal content. This is particularly relevant in quiet electric vehicles, where even weak tonal components can be perceptually salient [1,2,13,31].

A cover-integrated metamaterial can reduce the mobility of a radiating inverter or power-electronics panel without redesign of the electromagnetic source. The concept is attractive late in development, but cooling, sealing, electromagnetic compatibility, serviceability, and mechanical robustness must be preserved [24,31,46,67].

### 4.3. Gear Whine, Transmission Error, and Housing Radiation

Reduction gearboxes remain major tonal noise contributors in electric drivetrains. Gear whine is mainly driven by loaded transmission error, time-varying mesh stiffness, gear manufacturing deviations, tooth flank waviness, bearing dynamics, shaft compliance, and housing radiation. Because electric motors often operate over wide speed ranges, gear mesh frequencies and their harmonics may sweep through structural resonances of the gearbox or integrated e-drive housing [8,10,11,13].

This clear force-transfer-radiation chain makes the housing suitable for local resonators in walls, ribs, or inserts. The concept is useful only if it preserves bearing alignment, torque-reaction stiffness, sealing, lubrication, thermal behavior, fatigue resistance, and dimensional stability [8,13].

A practical future workflow should begin from order analysis or multibody drivetrain simulation rather than from an arbitrary target frequency. Critical gear orders and harmonics can be identified from transmission-error, mesh-force, and housing-response simulations. The metamaterial bandgap should then be tuned so that it overlaps with the radiating order under the most critical operating conditions. This order-targeted approach is a stronger EV-specific argument than a generic statement that metamaterials reduce vibration [6,8,10,11,13]. Deep learning-based inverse design of membrane-type metamaterials for gearbox-noise suppression provides a recent example of this excitation-to-geometry workflow [77].

### 4.4. Tire Cavity Resonance and Road-Noise Transfer

Tire–road interaction remains a dominant contributor to electric vehicle interior noise because the reduced powertrain masking exposes road-noise components more clearly. A particularly important low-frequency phenomenon is tire cavity resonance, often reported to be approximately 180–250 Hz depending on tire size, inflation pressure, load, and operating condition. The acoustic cavity inside the tire couples with the wheel, suspension, wheel arch, and body structure, generating structure-borne vibration that can enter the passenger compartment [1,6,7,13]. Adjacent semi-active inertial-suspension research also demonstrates that phase behavior and compensation influence vibration transfer through the suspension path; it is cited here as supporting vehicle-dynamics context rather than as direct metamaterial evidence [78].

The relatively stable target band and known transfer path make resonant wheel-arch treatment attractive compared with bulky low-frequency dampers. Vehicle-oriented metal AM demonstrators provide useful evidence, but production evaluation must include cabin response, added mass, corrosion, impact, contamination, attachment durability, and scalable alternatives to prototype manufacturing [7,32,44,45,46]. Additional automotive studies have examined wheelhouse liners, low-frequency vehicle treatments, and tire–pavement metasurfaces, broadening the evidence beyond a single wheel-arch demonstrator [79,80,81].

### 4.5. Auxiliary Systems: Electric Compressors, HVAC, Pumps, and Actuators

Auxiliary systems are more audible in electric vehicles than in many combustion engine vehicles because they are no longer masked by engine noise during low-speed or stationary operation. Electric compressors, coolant pumps, HVAC blowers, brake-system actuators, and other electromechanical subsystems may generate tonal or narrowband vibration. Their frequencies often lie in the low-to-mid-frequency region and may interact with local covers, brackets, or panels [1,6,13].

Component-specific membrane, cavity, or structural resonators may replace heavier encapsulation around compressors, pumps, or actuators, but tuning must remain stable across temperature, pressure, duty cycle, and aging [44,45,46].

### 4.6. Battery Enclosure Vibration and Large Lightweight Panels

Battery enclosures are EV-specific structures with strong relevance for low-frequency vibration control. They are large, relatively flat, and mechanically connected to the vehicle floor. Road input, chassis vibration, and local structural modes can excite the tray, cover, and internal support structures. In addition to NVH, vibration of the battery pack may influence electrical connections, module retention, seal durability, and long-term structural reliability [13,34,35].

Lattice and auxetic concepts can combine battery vibration control with support and crash functions, but performance depends strongly on host stiffness and damping. They must therefore be co-designed with materials, joints, thermal management, sealing, and crash requirements [34,35,67,68].

### 4.7. Body-Panel Booming, Door Radiation, and Interior Trim Structures

Large body panels, doors, floors, roofs, dash panels, and parcel shelves can radiate sound efficiently when excited by structure-borne vibration. In electric vehicles, these panels may contribute to booming, local tonal radiation, or secondary noise paths from road, drivetrain, or auxiliary sources. Conventional treatments such as damping pads and barriers remain useful, but they add mass and may be inefficient when the critical response is a narrow low-frequency mode [6,9,13,30,32,33].

Resonant patches, stamped patterns, polymer inserts, or trim-integrated elements can target door and body-panel velocity, but curvature, stiffeners, adhesive stiffness, crash requirements, water management, and corrosion can shift their response. Component testing is therefore essential [9,30,31,32,44,45]. Related studies on vehicle-door metamaterials and multi-band locally resonant body structures support the transferability of distributed resonators to finite automotive panels [82,83].

### 4.8. Psychoacoustic Importance of Tonal and Order-Related Components

EV NVH cannot be evaluated by overall sound pressure level (SPL) alone. A narrow tonal component can dominate subjective perception even when the broadband level is low. Psychoacoustic metrics such as loudness, sharpness, roughness, fluctuation strength, and tonality are therefore important when defining target frequencies for metamaterial design [1,2,3,4,40,41].

Metamaterial optimization should target the order or band with the greatest receiver-level annoyance, not necessarily the largest local vibration peak. Psychoacoustically weighted objectives can connect bandgap tuning to passenger comfort by incorporating tonal loudness, sharpness, roughness, or related indicators [1,2,3,4,36,37,40,41,47,48,49,50,51,52,53].

### 4.9. Implications for Order-Targeted Metamaterial Design

The most promising EV solutions are order- and path-targeted: locally resonant structures for housings, wheel arches, and panels; lattice or auxetic architectures for multifunctional enclosures; membrane and cavity concepts for covers and liners; and tunable systems when the order sweep exceeds passive bandwidth [6,7,8,9,23,26,27,30,31,32,33,34,35,36,37,47,48,49,50,51,52,53,55,64,65,66,67,69,70,72,73]. The mechanism-to-application decision matrix for the main low-frequency EV NVH problems is summarized in Table 2.

## 5. Automotive Applications and Component-Level Evidence

Automotive applications are the translation layer between metamaterial physics and EV NVH engineering. The most relevant locations are housings, covers, wheel arches, battery enclosures, body panels, and trim where tonal excitation couples to a lightly damped radiating or transmitting structure. This section therefore organizes the evidence by component and emphasizes transfer path, validation level, manufacturability, and unresolved risks [6,7,8,9,30,31,32,33,34,35].

**Figure 2:**
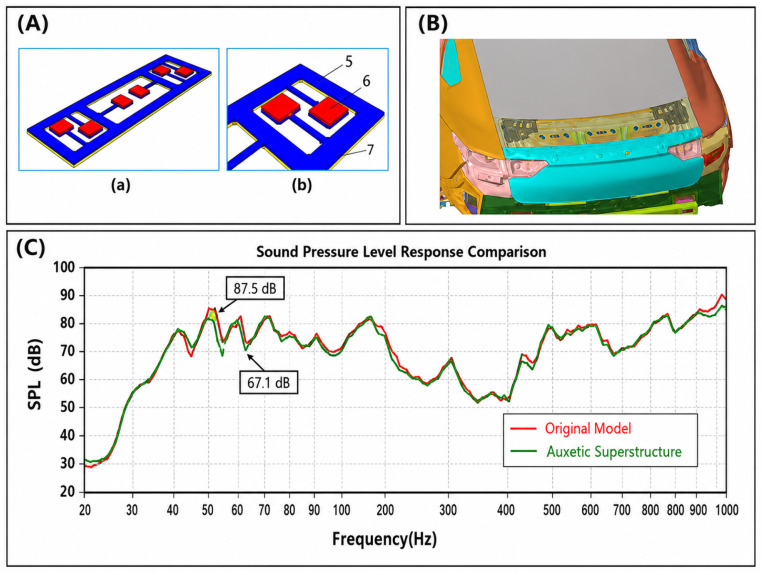
Representative vehicle-level application evidence reproduced from Liao et al. [80]: (**A**) designed locally resonant acoustic metamaterial and resonator unit, where (**a**) shows the designed locally resonant acoustic metamaterial and (**b**) shows the resonator unit; in subfigure (**b**), 5 denotes the metamaterial matrix plate, 6 the adjustable mass block, and 7 the damping layer (adapted from their Figure 16); (**B**) finite element integration of the metamaterial on the vehicle tailgate, with colors distinguishing the principal vehicle and structural model regions (their Figure 18); and (**C**) comparison of the driver-ear sound pressure level for the original state and the state with the acoustic metamaterial, showing the reduction around 35 Hz (their Figure 19). The panels were resized, cropped, and combined without altering the scientific content. © 2022 by the authors; reproduced and adapted under the Creative Commons Attribution 4.0 license.

For automotive implementation, vibroacoustic metamaterials should be positioned within a source–transfer–receiver framework rather than treated as isolated materials. Electric motors, gear meshing, inverter switching, tire–road interaction, and auxiliary systems act as excitation sources; housings, suspension interfaces, wheel arches, battery enclosures, doors, and body panels form the principal transfer and radiation paths. Figure 2 therefore replaces the earlier conceptual vehicle map with a representative published vehicle case that links resonator design, component integration, and receiver-level acoustic evidence. The broader evidence base also includes rear shock-tower patches, automotive silencers, electric-powertrain mounts, automotive glass, molded vehicle doors, pressure-relief valves, EV underbody resonator arrays, and power-electronics units [84,85,86,87,88,89,90,91].

A second distinction is the level of validation. Some studies remain at the numerical or unit-cell stage, while others have progressed to component demonstrators or vehicle-level tests. This distinction is essential because a metamaterial that performs well under periodic boundary conditions may lose effectiveness when installed on a curved, finite, damped, thermally loaded, and geometrically complex automotive component. The following sections therefore emphasize not only reported attenuation, but also the degree of experimental evidence and the unresolved implementation risks. Representative automotive applications, validation levels, reported relevance, and key implementation limitations are summarized in Table 3.

### 5.1. Gearbox and E-Drive Housings

Gearbox and e-drive housings are among the most important candidates for vibroacoustic metamaterial integration because they lie directly on the transfer path between drivetrain excitation and airborne radiation. Gear whine originates primarily from transmission error, time-varying mesh stiffness, gear-manufacturing deviations, and bearing-force transmission. Once these dynamic loads reach the housing, lightweight wall panels can radiate sound efficiently into the vehicle structure and cabin environment [8,10,11,13].

Amaral et al. linked lightweight gearbox-housing design with locally resonant features and radiated sound-power reduction [8]. The next step is to tune resonator placement from gear-order, bearing-force, modal, and radiation analyses so that the treatment becomes part of structural NVH design rather than a late add-on.

### 5.2. Electric Motor Casings and Integrated E-Drive Covers

Electric motor casings share several features with gearbox housings: they are compact, stiff, thermally loaded, and exposed to tonal excitations. Electromagnetic radial forces, torque ripple, slot/pole harmonics, inverter-related excitations, and bearing paths can all excite casing modes. Because these frequencies often vary with rotational speed, passive fixed-frequency metamaterials may be insufficient unless the design uses multiple resonances, graded resonators, or adaptive tuning [1,6,31].

Although mature motor-casing demonstrations remain limited, replaceable covers provide a practical development route for locally resonant or tunable treatments. Any solution must preserve thermal management, EMC, sealing, serviceability, and fail-safe behavior [31,36,37,49].

### 5.3. Wheel Arches and Tire Cavity Noise

Tire cavity noise is a strong example of a vehicle NVH problem that is well matched to a resonant metamaterial solution. The first acoustic resonance of the tire cavity often appears in the low-frequency range of approximately 180–250 Hz. This excitation is transmitted through the wheel, suspension, body attachment points, and wheel-arch structures before being perceived inside the cabin [6,7,13].

Sangiuliano et al. demonstrated vehicle-oriented metal AM wheel-arch resonators beyond a flat laboratory plate [7]. High-volume translation will require production-compatible geometries and validation of attachment, impact, contamination, corrosion, and temperature durability.

### 5.4. Battery Pack Casings and Large Lightweight EV Panels

Battery pack casings introduce a different design problem from tonal gearbox or tire-noise applications. They are large structural enclosures subjected to road-induced random vibration, local panel modes, sealing requirements, thermal management constraints, and crash-safety demands. Their large area makes them potential acoustic radiators, while their structural function prevents the use of weak or poorly validated acoustic add-ons [34,35,64,65].

Lattice and auxetic architectures can add vibration filtering and structural function to battery casings, but host-material damping strongly affects the result. Vibration reduction must therefore be validated together with crashworthiness, fatigue, fire safety, thermal expansion, sealing, electrical isolation, manufacturability, serviceability, and recyclability [34,35].

### 5.5. Body Panels, Doors, Dash Structures, and Parcel Shelves

Vehicle body panels and closures are natural targets for vibroacoustic metamaterials because they are often thin, lightly damped, and efficient acoustic radiators. Doors, dash panels, floors, roofs, tailgates, and parcel shelves may exhibit local resonances that amplify road, drivetrain, or auxiliary system excitation. Traditional damping sheets can reduce such radiation, but they add mass and may be inefficient when the problem is a very narrow low-frequency mode [6,9,30,32,33].

Door, dash, parcel-shelf, and body-panel demonstrators support targeted resonator integration, but curvature, attachment, coating, crash requirements, mechanisms, corrosion, repairability, and takt-time constraints determine production robustness [9,30,32,33].

### 5.6. Power-Electronics Covers and Auxiliary Enclosures

Electric powertrains include covers and enclosures for inverters, converters, electronics, pumps, and thermal management systems. These components may not be primary structural members, but they can radiate tonal noise when excited by switching-related forces, coolant pump orders, compressor vibration, or structure-borne e-drive paths [1,31].

Cover-integrated ribs, polymer resonators, membranes, or cavities provide a practical intermediate application because the main e-drive structure need not be redesigned. Their development must remain multi-objective: attenuation cannot compromise sealing, cooling, EMC, electrical safety, service access, or manufacturability [31].

### 5.7. Interior Trim, Acoustic Packages, and Cabin-Side Treatments

Membrane, coiled-space, labyrinthine, Helmholtz, and polymeric resonant systems can target cabin-side transmission in trim, undercovers, liners, and local inserts. Their low-frequency benefit is attractive, but flammability, odor, moisture, temperature, mounting, sealing, and recyclability remain production constraints [14,23,26,27].

In a production vehicle, interior metamaterials are unlikely to replace all porous absorbers or damping layers. A more realistic route is hybridization: porous material provides broadband mid- and high-frequency absorption, while a resonant meta-layer targets one problematic low-frequency tone or panel mode.

### 5.8. Active, Tunable, and Adaptive EV Meta-Structures

Active and tunable meta-structures adjust stiffness, damping, electrical impedance, or resonator state to follow motor and gear orders during acceleration and regeneration. Potential benefits must justify sensors, actuators, wiring, controls, EMC, calibration, diagnostics, and fail-safe validation [36,37,49].

The industrial barrier is substantial. Active metamaterials require sensors, actuators, wiring, control algorithms, calibration procedures, electromagnetic compatibility, diagnostics, and fail-safe validation. They should therefore first be considered for high-value tonal problems where passive solutions cannot cover the operating range without excessive mass or complexity.

To enable direct engineering comparison, Table 4 extracts the target frequency, reported attenuation, measurement metric, mass information, and validation level from representative component- and vehicle-level studies. “NR” denotes data not reported in a form that permits reliable extraction. Because the studies use different response metrics and baselines, the reported values should not be interpreted as a universal ranking. The reported dB/kg value is a descriptive screening index only and should not be interpreted as a linear physical efficiency measure because decibels are logarithmic.

### 5.9. Synthesis of Automotive Application Evidence

Table 4 shows that the largest peak reductions occur in controlled component tests, while vehicle-level effects are generally smaller but more directly relevant. Different metrics, baselines, and excitation conditions prevent a universal ranking; metamaterials should therefore be treated as targeted source–path–receiver tools rather than replacements for all classical treatments.

The evidence also reveals a systematic reporting gap: added mass, mass-normalized performance, and post-aging retention are frequently unavailable. Future automotive studies should therefore report the target band, response metric, baseline, added mass, attenuation per unit mass, validation level, and environmental condition as a minimum benchmarking set. Maturity remains strongly component-dependent: wheel-arch, cover, and local panel applications are closer to practical use, whereas gearbox housings and battery enclosures require deeper structural, durability, and safety validation.

## 6. Materials and Manufacturing Technologies

The practical value of vibroacoustic metamaterials in electric vehicles depends not only on their predicted bandgap or insertion-loss performance, but also on the material system and manufacturing route used to realize the architecture. This distinction is important because a metamaterial is not a conventional acoustic material with a single set of intrinsic properties. Its performance emerges from the interaction between material density, stiffness, damping, geometry, boundary conditions, resonator tuning, and attachment quality. For this reason, material selection and manufacturing technology must be considered as part of the vibroacoustic design problem rather than as later production details [6,7,8,26,27,28,29,67,68].

From an automotive perspective, the selected material system directly affects bandgap frequency, attenuation depth, structural stiffness, fatigue resistance, thermal stability, corrosion behavior, recyclability, and cost. A polymer resonator may be lightweight and easy to mold, but it may also be sensitive to humidity, creep, and temperature-dependent stiffness. A metallic resonator may provide better dimensional stability and fatigue resistance, but it may increase local mass or require more expensive manufacturing. A composite or lattice core may offer multifunctionality, but it introduces joining, repair, and end-of-life challenges. These trade-offs are especially severe in EV components, where NVH performance must be balanced with lightweighting, thermal management, crash safety, sealing, and lifecycle sustainability [34,46,55,64,65,66,67,68].

The following sections synthesize the principal material families and manufacturing technologies that appear in the reviewed literature. The emphasis is not only on what can be manufactured, but on whether the resulting system can realistically survive vehicle service conditions while maintaining its tuned vibroacoustic response.

### 6.1. Materials-Centric Design of Vibroacoustic Metamaterials

A materials-centric interpretation of vibroacoustic metamaterials starts from the observation that geometry and material behavior cannot be separated. The same resonator geometry will produce different bandgap frequencies when manufactured from aluminum, steel, polyamide, elastomeric polymer, carbon-fiber-reinforced polymer, or a hybrid sandwich assembly. The resonator stiffness, effective mass, damping, and local boundary compliance determine the resonance frequency and therefore the stop-band position. Even small changes in elastic modulus or adhesive stiffness can shift the attenuation band enough to reduce performance at the target order frequency [6,7,38].

This sensitivity creates a different design philosophy from conventional damping packages. A damping sheet may tolerate moderate property variation because its function is broadly dissipative. A locally resonant metamaterial, in contrast, must be tuned. Its design should therefore include material tolerances, manufacturing scatter, temperature dependence, and aging effects from the beginning. In EV applications, this is particularly important for speed-dependent motor and gear orders because a small frequency shift can move the stop band away from the most perceptible tonal component [1,6,7,8,33].

This point is important because material systems should not be treated as interchangeable substrates. Instead, the material family, architecture, and manufacturing route should be considered as a coupled design space. AI-assisted design becomes relevant in this context because the number of coupled variables quickly becomes too large for manual tuning alone [36,37,47,48,49,50,51,52,53].

### 6.2. Metallic Metamaterials

Metallic metamaterials are attractive for structural automotive applications because they can combine load-bearing capability with wave-control functionality. Aluminum alloys are especially relevant for EV gearbox housings, e-drive casings, battery enclosures, and lightweight structural covers. Their low density, corrosion resistance, castability, machinability, and compatibility with additive manufacturing make them suitable for integrated meta-structured components. Amaral et al. demonstrated that locally resonant concepts can be incorporated into lightweight gearbox-housing design, linking housing mass reduction with vibroacoustic attenuation [8]. Lee and Lee also showed the potential of mechanical metamaterial concepts in aluminum battery-casing configurations [34].

The main limitation of aluminum is its relatively low intrinsic damping. This means that the attenuation effect must be generated mainly by geometry, local resonance, or hybrid damping layers rather than by material damping alone. In a gearbox or motor housing, this can be beneficial because a low-damping metallic host allows clear resonator interaction, but it also means that structural modes may radiate efficiently if the metamaterial is mistuned or poorly located [8,13].

Steel-based systems remain relevant for stamped vehicle panels, door structures, reinforcements, brackets, and resonator inserts. Steel offers high stiffness, mature forming technology, good fatigue resistance, and a well-established automotive supply chain. Stamped steel resonators or periodic features may be more scalable than metal additive manufacturing for high-volume production. Their disadvantage is a higher density, which can reduce the lightweighting advantage if too much material is added. For this reason, steel-based VAMMs are more attractive when the resonant geometry can be created by forming existing sheet material rather than by adding separate masses [6,9,30].

Metal additive manufacturing broadens the design space by enabling compact resonator shapes, internal lattices, and complex cantilever or mass-spring geometries. Sangiuliano et al. used metal 3D-printed resonant elements for low-frequency tire-noise mitigation at the rear wheel arch, demonstrating the value of metallic AM for vehicle-oriented resonant metamaterials [7]. However, the cost, production rate, surface quality, residual stress, and fatigue behavior of additively manufactured resonators remain major barriers for series production [7,39].

### 6.3. Polymeric Metamaterials

Polymeric metamaterials are likely to be important for near-term automotive implementation because they are lightweight, relatively inexpensive, and compatible with scalable manufacturing routes such as injection molding, thermoforming, extrusion, and polymer additive manufacturing. Typical applications include wheel-arch liners, acoustic covers, trim panels, door-panel treatments, resonator arrays, and non-load-bearing secondary structures. In such components, the structural safety requirements are lower than in a gearbox housing or battery tray, making polymeric VAMMs easier to introduce industrially [6,30,31,32,33].

Polymer-based resonators also provide useful intrinsic damping. This can reduce excessive amplification near resonance and make the attenuation response less sharp. However, too much damping can reduce the depth of a locally resonant bandgap because the resonator no longer produces a strong out-of-phase response. This trade-off is important for battery and composite structures as well: a highly damped host can suppress resonator activity, while an undamped host may require additional treatment to control adjacent peaks [33,34].

The main risks of polymeric systems are temperature sensitivity, creep, moisture absorption, ultraviolet (UV) exposure, and long-term aging. A polyamide or elastomeric resonator that is correctly tuned at room temperature may shift after thermal cycling, humidity exposure, or prolonged mechanical loading. Therefore, polymeric metamaterials must be validated over the expected vehicle temperature and humidity range, not only under laboratory conditions [38,46].

A promising production route is to use additive manufacturing for prototype development and then redesign the successful architecture for injection molding or thermoforming. This route is consistent with several reviewed studies: complex resonator geometries were first demonstrated using 3D printing, while the final industrial concept would likely require a moldable, monolithic, or modular design [7,33].

### 6.4. Composite and Hybrid Material Systems

Composite and hybrid metamaterials are attractive when lightweight structural performance and vibroacoustic attenuation must be combined. Fiber-reinforced composites offer high specific stiffness and can be integrated into panels, covers, underbody structures, and battery enclosures. Hybrid systems may combine composite skins, metallic resonators, polymer damping layers, lattice cores, and acoustic cavities. Such configurations can provide multifunctional behavior, but they also increase manufacturing and recycling complexity [34,55,67].

A key issue is that composite hosts may have higher intrinsic damping than metallic hosts. This can be beneficial for broadband vibration reduction, but it may weaken sharply tuned local-resonance effects. The battery-casing results discussed earlier show that the same metamaterial architecture may perform differently in aluminum and composite configurations, highlighting the need to evaluate the host–resonator interaction rather than only the unit-cell geometry [34].

Hybrid systems are also sensitive to interfaces. Adhesive layers, co-cured joints, mechanical fasteners, insert molding, and overmolding can all alter the effective stiffness of the resonator boundary. Since the boundary condition is part of the dynamic system, joining technology becomes an acoustic design variable. This is a particularly important point for vehicle door panels, wheel-arch liners, and cover-integrated VAMMs, where attachment degradation could cause detuning, loss of attenuation, or secondary rattling noise [6,30,31,38].

### 6.5. Lattice, Cellular, Auxetic, and Pentamode Architectures

Lattice and cellular metamaterials derive their effective properties from unit-cell architecture. By changing cell size, strut diameter, topology, relative density, nodal geometry, and material distribution, designers can tailor stiffness, density, energy absorption, vibration transmission, and acoustic radiation behavior. This makes lattice structures especially relevant for multifunctional EV components such as battery trays, structural enclosures, support brackets, and crash-energy absorbers [34,64,65,66,67,68].

Auxetic structures are of particular interest because their negative Poisson ratio can improve indentation resistance, energy absorption, and deformation control. Lakes established the concept of negative-Poisson ratio foam structures, and later work expanded auxetic and flexible mechanical metamaterials toward engineered cellular materials [55,69,70]. In EV applications, auxetic lattices are promising for battery protection and vibration mitigation, but their technology readiness remains limited because crash, fatigue, temperature, and manufacturability constraints have not yet been fully resolved [34,35].

Pentamode and related architected elastic materials may become relevant when designers need to tune bulk and shear responses separately. Their immediate automotive use is still speculative, but they demonstrate the broader principle that elastic wave propagation can be controlled through architecture rather than material composition alone [39,72,73]. Accordingly, these structures are best interpreted as emerging candidates for future multifunctional EV components rather than as near-term production solutions. The principal material systems, their automotive applications, advantages, and implementation limitations are summarized in Table 5.

### 6.6. Additive Manufacturing as an Enabling but Transitional Technology

Additive manufacturing has enabled much of the recent progress in vibroacoustic metamaterials because it can realize internal cavities, slender resonators, lattice cores, dual-mode cantilevers, coiled acoustic paths, and topology-optimized geometries that are difficult or impossible to produce using conventional manufacturing. This is why several automotive-oriented demonstrators rely on metal or polymer AM, including wheel-arch resonators and low-frequency resonator arrays [7,33]. Complementary studies have demonstrated industrial-scale multi-bandgap sound-reduction materials, sheet-metal vibroacoustic meta-structures, dual-functional additively manufactured architectures, novel porous AM acoustic materials, and open-porous PLA structures developed for noise suppression in hybrid electric vehicles [92,93,94,95,96].

However, AM should be interpreted as both an enabler and a transitional development route. It is excellent for prototyping, geometry exploration, and low-volume specialized components, but it is not automatically suitable for high-volume automotive production. Cost, cycle time, surface quality, residual stresses, porosity, dimensional scatter, material anisotropy, and fatigue behavior can all influence the final vibroacoustic response [7,33,39].

For this reason, the most realistic industrial workflow may be: AM for early concept realization, test-based validation of the acoustic mechanism, redesign for scalable production, and final validation using the production-intent material and process. A resonator that works as a laser-sintered prototype may need geometric compensation when converted to injection molding or stamping because the material stiffness, damping, and tolerances will change [7,30,33,38].

**Figure 3:**
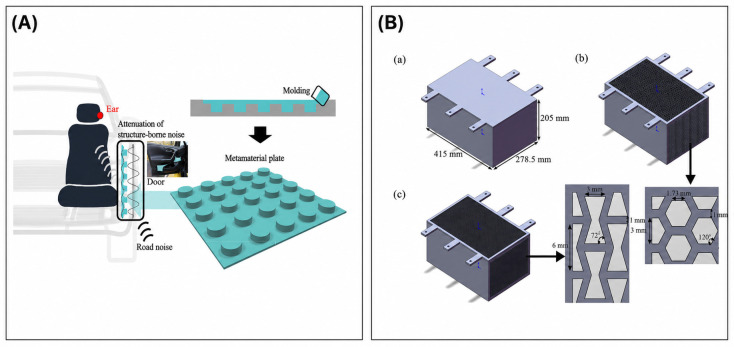
Representative door-related automotive vibroacoustic metamaterial concepts and applications. (**A**) Schematic illustration of a door-integrated metamaterial plate for attenuation of structure-borne road noise transmitted through the vehicle door, adapted from Tomita et al. [88], Figure 1. (**B**) Representative vehicle-door metamaterial demonstrator and design sequence: the internal subpanels (**a**–**c**) show the reference geometry, the metamaterial-integrated configuration, and the final patterned design together with the corresponding unit-cell details, adapted from Lee and Lee [34], Figure 1. Both source articles are distributed under the Creative Commons Attribution 4.0 license. The panels were cropped and enlarged to improve readability without altering the scientific content.

Figure 3 presents two representative production-oriented concepts from the reviewed literature. The molded periodic door-trim plate demonstrates how metamaterial functionality can be integrated through a single-material manufacturing process, while the lattice and auxetic battery pack casings illustrate multifunctional structural architectures. These examples provide a more direct link between geometry, component function, and production feasibility than a generic conceptual architecture chart.

### 6.7. Scalable Manufacturing Routes for Automotive Production

High-volume electric vehicles will require manufacturing technologies compatible with existing automotive production chains. Injection molding is attractive for polymeric resonator arrays, trim-integrated metamaterials, wheel-arch liners, acoustic covers, and non-load-bearing elements. Thermoforming may be suitable for larger polymer panels or liners. Sheet-metal stamping and forming are attractive for resonator patterns that can be integrated into body panels or brackets. Die casting and structural casting may be relevant for e-drive housings, gearbox casings, and aluminum support structures, provided that the meta-features do not compromise mold filling, structural strength, or dimensional stability [6,7,8,9,30,31,32,33,34,35,55,64,65,66,67,68,69,70].

The manufacturing route should be selected according to both geometry and function. A wheel-arch resonator may tolerate modular attachment if it is protected from debris and corrosion. A gearbox-housing meta-structure should preferably be integrated into the casting or housing architecture because loose or bonded parts inside an e-drive system would introduce unacceptable reliability risks. A battery tray lattice may need to be co-designed with crash and sealing requirements rather than added as an acoustic insert after the structural design is complete [8,34,35]. The evidence-supported comparison of manufacturing routes, including their scalability, geometric capability, material compatibility, and principal implementation constraints, is summarized in Table 6.

### 6.8. Material Degradation and Environmental Effects

Durability is one of the least resolved barriers to automotive vibroacoustic metamaterials (VAMMs) implementation. The same local resonance that produces strong attenuation can also create amplified relative motion, cyclic stress, and local strain concentration. Slender beams, thin lattice members, bonded masses, compliant layers, and membrane systems may therefore experience fatigue or property drift over service life. This problem is more severe in vehicles than in laboratory demonstrators because components are exposed to thermal cycling, humidity, salt spray, road debris, oils, vibration, impact loading, and assembly variation [38,39,46].

Temperature-induced detuning is especially important. A resonator tuned to a tire cavity peak or gear order at 20 °C may shift if the stiffness of a polymer, adhesive, elastomer, or membrane changes at low or high temperature. Moisture absorption can have a similar effect in polyamides and composites. Corrosion and surface degradation may affect metallic systems, particularly at joints, edges, and AM surfaces. Adhesive degradation can reduce boundary stiffness or cause partial debonding, which may eliminate the intended attenuation and introduce new rattling noise [38,46].

Future validation should therefore include beginning-of-life and end-of-life acoustic performance. A credible production development plan should combine modal and frequency response function (FRF) testing, temperature cycling, humidity exposure, vibration fatigue, corrosion or salt-spray testing (where relevant), and post-aging remeasurement of bandgap location and attenuation depth. Standards developed for vehicle environmental testing and material damping measurement provide a useful framework, although VAMM-specific automotive standards are still missing [42,43,44,45,46].

### 6.9. Quality Control and Manufacturing Scatter

Manufacturing scatter is not a secondary issue for resonant metamaterials. Small deviations in resonator length, thickness, mass, attachment stiffness, cavity volume, neck diameter, or lattice strut geometry can shift the resonance frequency. In a conventional structure, such deviations may be acceptable within general tolerance limits. In a locally resonant metamaterial, they can directly change the bandgap. Therefore, the relevant quality-control variable is not only dimensional accuracy, but also dynamic response [38].

A practical quality-control approach may include sample-based FRF testing, automated modal checks, laser vibrometry for prototype validation, and production monitoring of critical dimensions linked to resonance. For high-volume parts, it may be unrealistic to test every component acoustically. This increases the value of robust design: the bandgap should be wide enough, or the resonator distribution sufficiently varied, to tolerate realistic production scatter without losing the target attenuation [38,42,43,44,45].

### 6.10. Industrial Synthesis of Materials and Manufacturing Constraints

The reviewed evidence suggests that near-term industrial adoption will favor metamaterials that can be manufactured as monolithic, robust, and easily assembled components. Wheel-arch liners, covers, trim panels, and selected body-panel treatments are likely to mature earlier because they can use polymer molding, thermoforming, stamping, or modular attachment with lower structural risk. Gearbox housings and battery enclosures offer greater EV-specific value, but they require stronger proof of structural safety, fatigue resistance, thermal robustness, crash compatibility, and manufacturing repeatability [7,8,34,35].

A useful design rule is that the manufacturing process should be chosen before the final acoustic tuning is frozen. If the architecture is tuned using an AM prototype but later produced by injection molding or casting, the resonance frequency and damping behavior may change. Therefore, production-intent material properties and tolerances should be incorporated into the optimization loop as early as possible. This is a strong argument for AI-assisted and surrogate-model-based design workflows, where geometry, material, process, tolerance, and target frequency can be optimized together [36,37,47,48,49,50,51,52,53]. The principal production risks, their likely consequences, and the corresponding mitigation strategies are summarized in Table 7.

## 7. Modeling and Simulation Methods

The development of vibroacoustic metamaterials for electric vehicles requires modeling methods that connect local wave-control physics with full vehicle NVH behavior. A unit cell may show a clear bandgap under ideal Bloch–Floquet boundary conditions, but this does not automatically imply a cabin noise benefit. In practice, the metamaterial must interact with a finite, damped, curved, and imperfect vehicle component that is excited by order-related forces, road inputs, or auxiliary system loads. Therefore, modeling should be organized as a multi-scale chain rather than as a single unit-cell calculation [6,7,8,29,56,57,58,59].

For EV applications, the most useful simulation strategy links three levels: the unit-cell level, where resonance and dispersion behavior are defined; the component level, where insertion loss, vibration mobility, stress, and sound radiation are predicted; and the vehicle level, where the modified component is evaluated in the source–transfer–receiver chain. This distinction is essential because metamaterials generally modify transfer paths and radiating surfaces, while the excitation sources themselves are often governed by electromagnetic forces, gear mesh dynamics, tire–road interaction, or compressor orders [1,6,7,8,10,11,12,13].

The following sections synthesize the modeling methods most relevant to order-targeted vibroacoustic metamaterial design: unit-cell dispersion analysis, lumped and reduced-order resonator models, FE harmonic response, coupled structural–acoustic simulation, multibody drivetrain excitation modeling, transfer path analysis (TPA), statistical energy methods, surrogate models, and uncertainty-aware validation.

### 7.1. Unit-Cell Dispersion Analysis and Bloch–Floquet Modeling

Dispersion analysis is the classical starting point for periodic metamaterial design. The objective is to compute the relationship between frequency and wave vector and thereby identify pass bands and stop bands. For a periodic structure, Bloch–Floquet boundary conditions allow the infinite array to be represented by a single unit cell. This approach is computationally efficient and provides direct insight into the formation of Bragg or locally resonant bandgaps [16,17,18,19,20,29,56].

In its simplest form, the displacement field of a periodic elastic medium can be expressed as u(x+a)=u(x)exp(ika), where *a* is the lattice vector and *k* is the wave number. The resulting eigenvalue problem reveals the frequencies at which propagating solutions are absent. In phononic crystals, the bandgap is primarily associated with Bragg interference, whereas in locally resonant metamaterials the flat resonant branch of the internal oscillator interacts with the host structure and opens a subwavelength stop band [19,20,21,22].

For automotive use, the limitation of unit-cell dispersion analysis is that it assumes ideal periodicity, uniform material properties, and often infinite spatial extent. Real vehicle parts violate these assumptions: wheel arches are curved, gearbox housings contain ribs and bolted interfaces, door panels have cut-outs and reinforcements, and battery trays are constrained by sealing, crash, and thermal interfaces. Consequently, unit-cell analysis should be interpreted as a design-screening tool rather than as final proof of NVH performance [6,7,8,34].

### 7.2. Lumped-Parameter and Reduced Physical Models

Lumped-parameter models remain useful because many locally resonant metamaterials can be interpreted as distributed tuned vibration absorbers. A resonator can be approximated by an effective mass, stiffness, and damping, and its tuning frequency can be estimated from the classical relation $f_{0}=\frac{1}{2\pi }\sqrt{\frac{k}{m}}$. Such models provide rapid physical understanding of how resonator mass, beam stiffness, membrane tension, cavity volume, or neck geometry influence the target attenuation frequency [20,21,22,23,24,25,26].

The value of simplified models is especially high during early vehicle design, when full computer-aided design (CAD) geometry or validated damping data may not yet be available. They can be used to estimate whether a desired order frequency is physically reachable within available packaging space. For example, a tire cavity target near 200–250 Hz or a low-frequency body-panel mode may require a relatively compliant resonator, whereas a gear-whine-related housing feature at higher frequency can use a stiffer and more compact geometry [6,7,8,33].

However, lumped models must be used carefully. They may predict the approximate resonance frequency but often cannot capture finite-array effects, modal coupling, curved geometry, adhesive compliance, local stress concentration, or acoustic radiation. Therefore, they should support, not replace, finite element and experimental validation [6,7,8,30,31,32,33,34,38].

### 7.3. Component-Level FE Modeling

Finite element (FE) analysis is the main deterministic tool for component-level metamaterial design. It can represent the host structure, resonators, damping layers, lattice geometry, boundary conditions, and material anisotropy. In automotive applications, FE models are used to compare baseline and metamaterial-enhanced components in terms of modal behavior, mobility, vibration velocity, acceleration response, insertion loss, and structural stress [8,13,33,34].

For a linear structural model, the dynamic response can be expressed in matrix form as $M\ddot{x}+C\dot{x}+Kx=F(t)$, where *M*, *C*, and *K* are the mass, damping, and stiffness matrices; *x* is the displacement vector; and *F*(*t*) is the excitation force vector. In a metamaterial-enhanced component, local resonators modify all three dynamic properties: they add local inertia, introduce tuned stiffness, and may add damping through viscoelastic or material losses. This is why the same geometric architecture can perform differently when attached to aluminum, steel, polymer, or composite host structures [6,7,8,34,67,68].

Component-level FE modeling is particularly important for gearbox and e-drive housings. Gear mesh excitation and bearing forces do not act uniformly on a housing surface; they enter through specific shafts, bearings, and mounting interfaces. The relevant output is therefore not only the local bandgap of a unit cell, but the reduction in wall velocity and radiated sound power at the dominant transfer paths. Similar reasoning applies to wheel arches, battery trays, and power-electronics covers [7,8,31,34].

### 7.4. Harmonic Response, Random Vibration, and Insertion-Loss Simulation

Harmonic response analysis is used when the target excitation is tonal or order-related. The model is excited over a frequency sweep, and the response is compared with and without the metamaterial. This approach is suitable for gear whine, motor orders, inverter-related tones, compressor tones, and narrowband body-panel resonances. The main performance indicators are reductions in mobility, acceleration, velocity, strain energy, or sound radiation at the target frequency [6,7,8,31,33].

Random vibration analysis is more relevant for road-induced excitation and battery enclosure applications. Battery casings and underbody structures experience broadband road input rather than a single harmonic force. In such cases, the metamaterial should be assessed using transmissibility, RMS acceleration, stress response, and fatigue-relevant quantities. Lee and Lee showed that lattice and auxetic battery-casing concepts can strongly reduce vibration in numerical models, but production relevance still requires crash, thermal, fatigue, and experimental validation [34].

Insertion loss is a particularly useful metric because it measures the difference between the baseline and treated configuration under equivalent excitation. This metric is preferable to reporting only a bandgap frequency because it directly describes the practical benefit of adding the metamaterial to a finite component [7,8,30,31,32,33,34].

### 7.5. Structural–Acoustic Coupling and FEM-BEM Workflows

Because many EV NVH problems are ultimately evaluated by radiated sound or cabin sound pressure, structural simulation must often be coupled to acoustic prediction. A metamaterial may reduce local vibration without producing a proportional acoustic benefit if the treated region is not an efficient radiator or not located on a dominant transfer path. Conversely, a moderate reduction in wall velocity on a highly radiating panel may produce a meaningful sound pressure reduction [13].

Coupled finite element method–boundary element method (FEM–BEM) workflows are widely used for sound radiation from housings, covers, body panels, and enclosures. The FE model calculates structural vibration, usually surface velocity or normal acceleration, and the boundary element model predicts sound pressure, sound power, or radiation efficiency. For gearbox and e-drive housings, this workflow is important because tonal excitation from gears or motors may excite housing modes that radiate efficiently into the surrounding structure or cabin [8,10,11,12,13].

For cabin-side applications, the acoustic cavity may need to be represented as well. A door, dash panel, floor, or battery tray can interact with the enclosed vehicle cabin volume. In such cases, structural–acoustic coupling helps determine whether the metamaterial reduces the response at the listener position or only changes the local vibration pattern [9,13,30,31].

### 7.6. Multibody Dynamics and Excitation-Source Modeling

Metamaterial design should not begin from an arbitrary target frequency. In EVs, the target band should be derived from the actual excitation source and its transfer efficiency. For gearboxes, multibody dynamics and gear-contact models can estimate transmission error, time-varying mesh stiffness effects, gear mesh forces, bearing reactions, shaft dynamics, and housing excitation. For electric motors, electromagnetic simulation can provide radial and tangential force harmonics that excite the stator and housing. These excitation models define the order content that the metamaterial must address [8,10,11,12].

A practical workflow is to use drivetrain simulations to identify critical operating points and order lines, then map the resulting forces into a structural model of the housing or vehicle component. The metamaterial is then tuned to reduce the response at frequencies that are both strongly excited and efficiently radiated. This prevents the common error of tuning a resonator to a frequency that is physically interesting but not dominant in the vehicle sound response [8,10,11,12,13].

This source-informed approach is especially relevant for speed-dependent EV noise. Gear mesh orders and motor electromagnetic orders sweep across frequency during acceleration. A fixed passive metamaterial can only cover a limited band, so the simulation workflow must identify the most critical operating condition, the most perceptually relevant order, or the frequency range where several operating paths overlap [1,2,3,4,6,7,8].

### 7.7. Transfer Path Analysis and Order-Based Vehicle Integration

TPA provides the system-level framework needed to evaluate metamaterials in vehicles. Since metamaterials typically modify a transfer path or radiating component rather than eliminating the source, their benefit should be measured in terms of the change in the source–transfer–receiver chain. For example, a wheel-arch resonator modifies the road-noise path from tire and suspension excitation to the cabin. A meta-structured gearbox housing modifies the path from gear mesh forces to housing radiation. A battery tray lattice modifies the transmission of road-induced vibration into the floor structure [6,7,8,13].

TPA is also useful for ranking candidate locations. A metamaterial should be placed where structural intensity, mobility, modal participation, or panel contribution analysis indicates that vibration energy is actually transmitted. Without this step, even a well-designed unit cell may be located on a secondary path and produce little cabin benefit [6,7,8,9,13].

Order-based integration is the EV-specific extension of this idea. The target should be specified not only as a frequency but as a source order, operating condition, and transfer path. This makes the design more transparent: for example, a wheel-arch treatment may target the first tire cavity resonance, while an e-drive housing treatment may target the dominant gear mesh order or electromagnetic force harmonic at a critical speed range [1,6,7,8].

### 7.8. Statistical Energy Analysis and Hybrid Mid-Frequency Models

Statistical energy analysis is less central for low-frequency metamaterial design than deterministic FE modeling because metamaterials often operate through localized resonances and wave phenomena. However, statistical energy analysis (SEA) and hybrid FEM-SEA approaches become useful when evaluating larger vehicle structures or higher-frequency airborne sound paths. They can support system-level sound-package development and help estimate how a local metamaterial treatment interacts with broader vehicle acoustic behavior [13,14].

A likely practical route is to use deterministic FE models for the treated component and hybrid methods for the full vehicle context. For example, an e-drive housing with locally resonant ribs may be modeled in detail, while surrounding body and cabin subsystems are represented in a reduced or statistical framework. This allows the detailed metamaterial physics to be retained without making the full vehicle model computationally prohibitive [13,14,29].

### 7.9. Reduced-Order Models and Homogenized Descriptions

Detailed metamaterial FE models can become computationally expensive because they contain many repeated resonators, thin beams, lattice struts, or acoustic cavities. Reduced-order models and homogenized descriptions therefore play an important role in practical design. A repeated resonator array can sometimes be represented by frequency-dependent effective mass, stiffness, damping, or impedance, allowing system-level simulations to include the metamaterial effect without meshing every small feature [26,27,28,29,36,37,47,48,49,50,51,52,53].

Homogenization must be applied cautiously because metamaterials are dispersive and strongly frequency-dependent. A single constant material property cannot represent a locally resonant bandgap over a broad frequency range. The most useful reduced descriptions are therefore frequency-dependent and validated against detailed FE or experimental results. In automotive computer-aided engineering (CAE) workflows, such reduced representations may become essential if VAMMs are to be included in full-vehicle models [26,27,28,29,36,37,47,48,49,50,51,52,53].

### 7.10. Uncertainty, Sensitivity, and Model Updating

Uncertainty analysis is not optional for resonant metamaterials. Small changes in geometry, material stiffness, damping, adhesive compliance, boundary condition, temperature, or manufacturing tolerance can shift the attenuation band. This is especially critical when the target is a narrow tonal order. Studies on stop-band uncertainty highlight the need to consider production scatter and parameter variation before claiming robust automotive performance [38].

Sensitivity studies should identify which parameters most strongly control the resonance frequency and attenuation depth. For beam-type resonators, this may include beam length, thickness, end mass, and attachment stiffness. For Helmholtz or labyrinthine resonators, cavity volume and neck geometry are critical. For lattice structures, strut diameter, unit-cell topology, relative density, and node geometry may dominate the response. These sensitivities should feed directly into tolerancing and quality-control plans [20,21,22,23,24,25,26,34,38,53].

Model updating is the bridge between simulation and experiment. Prototype FRF measurements, modal tests, laser vibrometry, and acoustic measurements can be used to adjust material damping, boundary stiffness, and resonator tuning in the numerical model. This is particularly important because many damping and interface properties are difficult to predict from nominal material data alone [42,43,44,45].

### 7.11. Proposed Simulation Workflow for Order-Targeted EV Metamaterial Design

A credible EV workflow should start from the NVH problem, not from the unit cell. First, the dominant tonal or low-frequency issue should be identified using operational measurement, order analysis, psychoacoustic evaluation, or drivetrain simulation. Second, the main transfer path and radiating component should be ranked using modal analysis, transfer path analysis, structural intensity, or panel contribution analysis. Third, a metamaterial concept should be selected according to frequency range, packaging, material, durability, and manufacturability constraints. Only then should unit-cell tuning, component-level FE simulation, and physical prototyping begin.

This workflow also clarifies the role of AI-assisted design. ML and surrogate models are most useful after the target frequency, component location, design constraints, and manufacturing route are known. Otherwise, optimization may produce a mathematically impressive architecture that does not solve the actual vehicle NVH problem. The modeling workflow should therefore connect source identification, transfer path ranking, resonator design, manufacturing constraints, and validation in a single loop [36,37,47,48,49,50,51,52,53]. The principal modeling and simulation methods, their roles in the development workflow, and their main strengths and limitations for EV vibroacoustic metamaterial applications are summarized in Table 8.

The proposed order-targeted simulation workflow, from excitation identification and transfer-path analysis to metamaterial optimization and validation, is summarized in Table 9.

## 8. Experimental Validation and Characterization Methods

Experimental validation is a decisive step in the development of vibroacoustic metamaterials for electric vehicles. Unit-cell calculations and FE models can predict resonances, dispersion curves, and attenuation bands, but they cannot by themselves prove that a meta-structure will remain effective after integration into a real vehicle component. Automotive structures are finite, curved, locally reinforced, bolted, bonded, thermally loaded, and exposed to manufacturing scatter. These conditions can shift resonance frequencies, reduce attenuation depth, or introduce new local amplification mechanisms. Therefore, experimental validation must be treated as a central part of the design process rather than as a final confirmation step [6,7,8,9,30,31,32,33,34,38,39,40,41,42,43,44,45,46].

The validation problem is particularly important for locally resonant metamaterials because their performance is based on frequency-selective dynamic interaction. A small change in beam thickness, adhesive stiffness, damping-layer modulus, polymer aging, or boundary condition can move the attenuation band away from the target gear order, tire cavity resonance, or motor harmonic. For this reason, a credible validation strategy should verify not only the beginning-of-life attenuation level, but also the robustness of the bandgap after thermal, mechanical, and environmental exposure [38,39,46].

### 8.1. Validation at Unit-Cell, Coupon, and Subcomponent Level

The first validation level usually focuses on the unit cell, resonator, or small coupon. The objective is to verify whether the manufactured geometry produces the resonance frequency, modal shape, and local dynamic behavior predicted by analytical or numerical models. For beam-type resonators, this may involve impact-hammer testing, shaker excitation, accelerometer measurements, or laser vibrometry. For membrane and acoustic cavity systems, impedance-tube measurements can be used to identify absorption, reflection, and transmission characteristics under standardized one-dimensional conditions [23,26,27,42].

Coupon-level testing is useful because it isolates the metamaterial mechanism from the complexity of the complete vehicle. However, it also introduces a risk of overestimating applicability. A bandgap demonstrated on a simply supported plate or a small flat panel may change substantially when the same resonator is attached to a curved door structure, a ribbed gearbox housing, or a wheel arch with local curvature and spot-welded interfaces. Coupon tests should therefore be interpreted as mechanism validation, not as vehicle-level performance evidence [6,7,8,9,30,31,32].

### 8.2. Experimental Modal Analysis and Frequency Response Functions

Experimental modal analysis provides the basic dynamic information required to understand both the host structure and the resonant metamaterial. Natural frequencies, mode shapes, damping ratios, and modal participation factors can be extracted from controlled excitation tests. For automotive meta-structures, modal analysis is especially useful for checking whether the resonators are tuned to the correct frequency range and whether the host structure exhibits vibration modes that can effectively couple with the resonators [8,13,30,31].

Frequency response functions (FRFs) are often the most direct indicator of metamaterial effectiveness. By comparing mobility, acceleration, velocity, or transfer functions before and after the integration of the meta-structure, the engineer can quantify insertion loss and attenuation depth. In locally resonant systems, the target band usually appears as a strong reduction in response amplitude, sometimes accompanied by anti-resonance behavior and phase changes around the resonant frequency. FRF-based validation is therefore suitable for gearbox housings, wheel-arch resonators, door-panel demonstrators, and battery enclosure prototypes [6,7,8,9,30,31,32,33,34].

A practical difficulty is that resonant metamaterials can create local changes in the response that are not fully captured by a small number of accelerometers. If the measurement grid is too sparse, local resonator activation, wave localization, or bypass paths may be missed. Therefore, FRF measurements should ideally be combined with full-field vibration measurements whenever the structure is spatially complex.

### 8.3. Scanning Laser Doppler Vibrometry and Wave-Field Characterization

Scanning laser Doppler vibrometry (SLDV) is one of the most powerful tools for validating vibroacoustic metamaterials because it provides non-contact, high-resolution vibration maps. Unlike point accelerometers, SLDV can visualize bending-wave propagation, local resonator motion, wave attenuation, and spatial energy redistribution across the treated structure. This is particularly valuable when the design objective is not simply to reduce a single point response but to prevent wave propagation toward radiating panels [7,8,30,31].

Full-field measurements can also support experimental dispersion analysis. By exciting a periodic or quasi-periodic specimen and measuring the propagating wave field, the experimental wavenumber–frequency relationship can be estimated and compared with Bloch–Floquet or FE predictions. This provides stronger evidence than point FRFs alone because it confirms the actual wave-control mechanism behind the observed attenuation. In automotive applications, such measurements are useful for wheel-arch panels, door demonstrators, lightweight covers, and simplified e-drive housing sections.

### 8.4. Acoustic Measurements, Sound Power, and Psychoacoustic Assessment

Structural attenuation does not automatically guarantee a meaningful cabin-noise benefit. A metamaterial may strongly reduce local vibration at one point while producing only a small change in radiated sound if the treated region is not acoustically dominant. For this reason, vibration measurements should be complemented by acoustic measurements, including microphone-based sound pressure level, sound intensity, sound power estimation, and microphone-array source localization [13,43].

For EV applications, psychoacoustic assessment is also important. Tonal components such as gear whine, inverter-related tones, and motor orders may remain disturbing even at moderate SPL values. Therefore, validation should not rely only on broadband dB reduction. Metrics such as loudness, sharpness, tonality, roughness, and fluctuation strength can provide a better link between physical attenuation and perceived sound quality [1,2,3,4,40,41]. This is especially relevant when a metamaterial is designed to suppress a narrow order rather than to reduce broadband noise. The principal experimental validation methods, their measurement objectives, and their relevance to automotive vibroacoustic metamaterial development are summarized in Table 10.

### 8.5. Component-Level Validation for EV-Relevant Structures

Component-level validation is the first stage at which a metamaterial concept can be evaluated under realistic geometry and boundary conditions. For gearbox and e-drive housings, this means exciting the housing through bearing locations, internal shaft-support regions, or representative gear mesh force inputs, and then measuring wall velocity and radiated sound power. The validation question is not only whether a local resonator moves at the intended frequency, but whether the housing mobility between excitation input and radiating panel is reduced [8].

For wheel-arch applications, component validation should reproduce the structure-borne transfer path associated with tire cavity resonance and road excitation. The work on metal 3D-printed wheel-arch resonators is important because it moves beyond an idealized beam or plate and demonstrates a metamaterial treatment within a vehicle-relevant structure [7]. However, the same application also illustrates the need for fatigue, corrosion, attachment, and manufacturing-cost assessment before industrial adoption.

For battery pack casings, the validation requirements are broader. A battery enclosure meta-core must be evaluated not only for vibration attenuation, but also for stiffness, crashworthiness, sealing, thermal behavior, and fatigue. Numerical studies suggest strong vibration-reduction potential for lattice and auxetic structures, especially in metallic casings, but the reviewed evidence remains mainly simulation-based [34,35]. Experimental testing of battery-casing metamaterials should therefore include random vibration, shock loading, thermal cycling, and post-test modal or FRF reassessment.

For doors, dash panels, power-electronics covers, and interior panels, component validation can focus on radiating panel velocity and local acoustic contribution. These components are often attractive early implementation targets because the metamaterial can be integrated as a secondary structure, cover, liner, or patch rather than as a primary load-bearing part [9,30,31,32].

### 8.6. Vehicle-Level Testing and Transfer Path Validation

Vehicle-level validation is ultimately necessary because the cabin response depends on the complete source–transfer–receiver chain. A local vibration reduction does not automatically translate into lower perceived cabin noise if other parallel paths dominate. For this reason, metamaterial-treated components should be evaluated using order tracking, operational transfer path analysis, chassis dynamometer tests, road tests, and controlled excitation measurements whenever possible [1,6,7].

The most suitable vehicle-level tests depend on the target source. Gear-whine applications require speed sweeps and torque-dependent operating conditions to determine whether the metamaterial stop band overlaps with the problematic gear mesh order across the relevant speed range. Tire cavity applications require repeatable tire–road or drum excitation and careful separation of tire, suspension, and body contributions. Power-electronics and motor-order applications require operating conditions that reproduce the electromagnetic force orders and inverter-related tonal components.

A useful validation metric is insertion loss, defined as the difference between the untreated and treated response under equivalent excitation and boundary conditions. For EV metamaterials, insertion loss should be reported both locally, for example, panel velocity reduction, and at receiver level, for example, cabin SPL, sound power, or psychoacoustic metric reduction. This dual reporting prevents overinterpretation of local structural improvements that do not affect passenger perception.

### 8.7. Durability, Aging, and Environmental Robustness

Durability validation is one of the most underdeveloped aspects of automotive vibroacoustic metamaterials. Local resonators frequently operate near their resonance frequency, which may amplify relative displacement and cyclic stress. Slender beams, lattice members, adhesive joints, and polymer springs may therefore be exposed to fatigue mechanisms that are not present in conventional homogeneous acoustic treatments [38,39].

Environmental robustness is equally important. Automotive components experience temperature cycling, humidity, salt spray, road debris, oils, mechanical shock, and continuous broadband vibration. These conditions can change material stiffness and damping, particularly in polymers, viscoelastic layers, and adhesives. Since resonance frequency depends directly on stiffness and mass, even moderate property changes can detune the metamaterial from the target order or panel mode [44,45,46].

A production-relevant validation plan should therefore include beginning-of-life and end-of-test measurements. Modal parameters, FRFs, insertion loss, and acoustic performance should be measured before and after aging. A resonator that provides strong initial attenuation but shifts outside the target frequency band after thermal cycling cannot be considered robust for vehicle deployment. Similarly, debonding or partial attachment failure may not only reduce performance but also introduce secondary rattling noise. The proposed validation roadmap, covering material characterization, component-level testing, environmental durability assessment, and vehicle-level verification, is summarized in Table 11.

### 8.8. Model Updating, Uncertainty Quantification, and Data-Driven Validation

Experimental validation should feed back into the simulation workflow. Differences between measured and predicted resonance frequencies can be used to update material parameters, boundary stiffness, damping values, and adhesive-layer models. This is especially important for metamaterials because their attenuation depends on narrow dynamic features rather than on slowly varying bulk properties. Model updating should therefore be performed before the design is scaled from a coupon to a vehicle component.

Uncertainty quantification is also essential. Manufacturing scatter in resonator dimensions, local stiffness, or attachment quality should be propagated through the simulation model to estimate the expected variation in stop-band frequency and attenuation depth. The literature on stop-band uncertainty shows that resonant systems can be highly sensitive to parameter scatter [38]. In an automotive context, this means that the design should not be tuned exactly to a single nominal frequency but should provide sufficient bandwidth or robustness to cover production and operating variability.

Data-driven validation can support this process. Measured FRFs, laser vibrometry maps, acoustic spectra, order-tracking results, and manufacturing inspection data can be used to train surrogate models or update digital twins. In the longer term, such workflows could allow metamaterial designs to be continuously improved using data from simulation, prototype testing, and operational vehicle measurements [36,37,47,48,49,50,51,52,53].

### 8.9. Recommended Validation Chain for Automotive Deployment

A practical validation chain for EV vibroacoustic metamaterials should follow a V-cycle logic. The left side of the V begins with the target NVH problem, order content, structural transfer path, and unit-cell design. The bottom of the V is prototype manufacturing and mechanism validation. The right side of the V climbs back toward component, subsystem, and vehicle-level performance confirmation. This logic helps prevent a common weakness in metamaterial studies: proving the existence of a bandgap without demonstrating that it solves a vehicle-relevant NVH problem.

Experimental validation is the main link between metamaterial physics and vehicle implementation. The reviewed literature demonstrates that the physical mechanisms are mature enough for targeted component studies, but the evidence base remains thinner for long-term durability, standardized benchmarking, and full-vehicle performance. Closing this validation gap is one of the most important conditions for the transition from promising laboratory metamaterials to production-relevant EV NVH solutions.

## 9. Artificial Intelligence and Data-Driven Design of Vibroacoustic Metamaterials

AI and data-driven optimization are useful because metamaterial design is nonlinear, multi-parameter, and multi-objective. The attenuation behavior of a metamaterial depends on geometry, material properties, resonator topology, damping, boundary conditions, manufacturing tolerances, and target operating frequencies. A conventional trial-and-error workflow based only on repeated FE simulations is therefore too slow for automotive development, particularly when the design must be aligned with speed-dependent motor orders, gear mesh harmonics, tire cavity resonances, and vehicle-level transfer paths [36,37,47,48,49,50,51,52,53].

For EVs, AI is useful for more than fast unit-cell optimization. Its real potential is the connection of several information layers that are normally handled separately: drivetrain-order maps, structural modal response, transfer path ranking, manufacturing constraints, material aging, and acoustic perception. A data-driven workflow can therefore support the transition from generic metamaterial concepts toward order-targeted, manufacturable, and experimentally validated EV components [6,7,8,9,30,31,32,33,34,35,36,37,47,48,49,50,51,52,53].

### 9.1. Why AI Is Relevant for EV Metamaterial Design

The design space of vibroacoustic metamaterials is large even for a simple periodic resonator plate. Beam length, thickness, mass distribution, cell spacing, damping-layer modulus, resonator orientation, host-panel stiffness, and attachment properties can all shift the bandgap location and attenuation depth. When the concept is extended to an e-drive housing, wheel arch, battery enclosure, or power-electronics cover, the number of variables increases further because the resonators must be placed on a finite and irregular structure rather than on an ideal infinite lattice [6,7,8,9,30,31,32,33].

AI methods are useful in this context because they can learn the mapping between design variables and dynamic performance from simulation or experimental datasets. Once trained, a surrogate model can evaluate candidate geometries in milliseconds rather than requiring a full FE calculation for each case. This makes it possible to perform broad design-space exploration, uncertainty screening, and multi-objective optimization within development time scales that are realistic for automotive engineering [47,48,49,50,51].

However, AI should not be treated as a replacement for physical modeling. Metamaterial behavior is governed by resonances, wave propagation, damping, and boundary coupling. Data-driven models that ignore these physical constraints can produce geometries that appear optimal numerically but fail after manufacturing or integration. The strongest approach is therefore a hybrid one: physics-based simulation generates reliable training data, ML accelerates exploration, and experiments update or correct the model [36,37,47,48,49,50,51,52,53].

### 9.2. Surrogate Modeling for Fast Bandgap and Performance Prediction

Surrogate modeling is currently one of the most practical AI tools for metamaterial design. A surrogate model approximates the relationship between input variables and output performance metrics. Typical inputs include lattice constants, beam dimensions, resonator masses, material stiffness, damping parameters, and local geometry descriptors. Typical outputs include resonance frequency, bandgap boundaries, attenuation depth, insertion loss, transmission loss, or radiated sound power reduction [47,48,49,50,51].

Several machine learning algorithms are suitable for this task. Gaussian Process Regression can be useful when uncertainty estimation is important. Random Forest and Extra Trees models are robust for mixed discrete–continuous design spaces and can provide feature-importance indicators. Support Vector Regression can perform well with smaller datasets, while artificial neural networks are effective when the design space is highly nonlinear and sufficient training data are available [47,48,49,50,51].

In an EV workflow, surrogate models can be trained on unit-cell dispersion simulations, harmonic response results, or component-level insertion-loss data. The trained model can then be used to rapidly scan candidate resonator geometries for a target gear order or tire cavity frequency. This is especially useful during early design stages, when the engineer must decide whether a target frequency can be reached without excessive mass, packaging volume, or structural weakening. The principal artificial intelligence methods, their roles in vibroacoustic metamaterial design, and their relevance to electric vehicle NVH applications are summarized in Table 12.

### 9.3. Inverse Design for Targeted Frequency Attenuation

Inverse design reverses the conventional metamaterial design process. Instead of proposing a geometry and then checking its bandgap, the engineer specifies the desired performance first, such as a stop band centered at 230 Hz, a minimum insertion loss around a gear mesh order, or a maximum allowed mass. The algorithm then searches for geometries that satisfy these requirements [48,49,50,51].

This approach is particularly relevant for EV NVH because the target frequencies are often known from order analysis. Gear mesh frequency, motor electromagnetic orders, inverter-related tonal components, tire cavity resonance, and auxiliary compressor tones can be extracted from measurement or simulation. Once these target bands are known, inverse design can generate resonator dimensions, lattice parameters, or placement layouts that align with the critical frequencies [6,7,8,33,36,37,47,48,49,50,51,52,53].

A robust inverse-design workflow should include constraints from manufacturing and durability. Without such constraints, optimization may produce extremely thin beams, sharp internal corners, or fragile lattice members that are numerically effective but unsuitable for automotive lifetime loads. For this reason, practical inverse design should include minimum feature sizes, allowable stresses, fatigue margins, temperature-dependent material behavior, and assembly constraints [38,39,44,45,46].

### 9.4. Topology Optimization and Multi-Objective Meta-Structure Design

Topology optimization is closely related to AI-assisted design because it automatically distributes material within a design domain to satisfy objective functions. In metamaterial applications, objectives may include maximizing the relative bandgap width, centering a stop band on a target frequency, minimizing vibration transmission, reducing radiated sound power, or preserving stiffness while reducing mass [36,37,52,53].

For EV components, topology optimization should be multi-objective. A gearbox housing cannot be optimized only for acoustic attenuation because it must also preserve bearing alignment, stiffness, thermal performance, lubrication clearances, manufacturability, and fatigue resistance. A battery enclosure cannot be optimized only for vibration isolation because it must also meet crash, sealing, thermal, and safety requirements. The same argument applies to wheel arches, power-electronics covers, and structural panels [8,31,34,35].

Recent meta-shell and resonator-placement optimization studies illustrate the importance of selecting both the resonator geometry and its spatial distribution. A dense uniform resonator array may provide strong attenuation but can add unnecessary mass. Discrete placement optimization can identify only those positions where resonators have high modal or transfer path influence, thereby improving the attenuation-to-mass ratio [37].

### 9.5. Generative AI and Automated Architecture Discovery

Generative AI methods are beginning to influence acoustic and elastic metamaterial design. Variational autoencoders, generative adversarial networks, diffusion-type models, attention-based networks, and other latent-space methods can generate new candidate architectures rather than only optimizing predefined parameter sets [48,49,50,51].

For vibroacoustic metamaterials, this is attractive because high-performing unit cells are often geometrically unintuitive. Human designers tend to work with familiar beams, membranes, masses, or cavities, whereas generative models can explore irregular cellular, folded, hierarchical, or multi-resonant geometries. These designs may achieve wider bandgaps, lower target frequencies, or better stiffness-to-mass ratios than manually parameterized concepts [50,51].

Nevertheless, generative AI remains early-stage for automotive implementation. Many generated designs are validated only numerically, and manufacturability is not always included in the learning process. Future EV-oriented generative design should include process constraints such as additive manufacturing limits, injection-molding draft angles, sheet-metal formability, minimum wall thickness, bonding surfaces, and quality-control tolerances.

### 9.6. Data Sources for EV-Specific AI-Assisted Metamaterial Development

A key difference between generic metamaterial design and EV-specific metamaterial design is the diversity of available data. Electric vehicle NVH development produces drivetrain simulations, electromagnetic force spectra, multibody gear-contact results, FE modal models, acoustic simulations, order-tracking measurements, production-quality data, and vehicle-level validation measurements. AI-assisted design should make use of this broader data environment rather than relying only on isolated unit-cell simulations.

For gearbox and e-drive applications, useful data sources include gear mesh orders, transmission error simulations, time-varying mesh stiffness, bearing-force spectra, housing mobility, ODS, and radiated sound power. For tire-noise applications, relevant data include tire cavity resonance frequency, suspension transfer paths, wheel-arch structural intensity, and cabin SPL around the first cavity mode. For battery enclosures, random vibration spectra, enclosure modes, strain data, and thermal or crash constraints should be included [6,7,8,9,30,31,32,33,34,35].

Manufacturing data can also improve robustness. Dimensional inspection, additive manufacturing deviations, injection-molding shrinkage, adhesive-layer thickness, and material-property scatter can be included as input variables. This allows the AI model to predict not only nominal performance, but also production variability and detuning risk [38,44,45,46]. The principal data sources, input features, target variables, and validation outputs for EV-specific AI-assisted metamaterial development are summarized in Table 13.

### 9.7. AI-Enabled Order-Targeted EV Metamaterial Workflow

The most promising use of AI in this field is an order-targeted design workflow. In the first step, the dominant EV excitation orders are identified from measurements or simulations. These may include gear mesh orders, electromagnetic motor orders, inverter-related tonal components, tire cavity resonances, or compressor orders. In the second step, transfer path and modal analyses identify which structures radiate or transmit these frequencies most efficiently [1,6,7,8,10,11,12,13].

In the third step, a surrogate model or inverse-design algorithm proposes candidate metamaterial geometries that create a stop band around the target frequency. In the fourth step, component-level simulations evaluate whether the proposed geometry reduces wall velocity, transfer mobility, or radiated sound. In the fifth step, optimization filters the candidates based on mass, stiffness, packaging, fatigue, and manufacturing constraints. Finally, experimental validation updates the model and closes the design loop.

This workflow is more realistic than designing a generic low-frequency metamaterial in isolation. The target is not simply to create a bandgap, but to create a bandgap that overlaps with a vehicle-relevant excitation and modifies a transfer path that actually contributes to cabin noise. This distinction is essential for industrial relevance.

### 9.8. Explainability, Physical Consistency, and Trustworthiness

A recurring limitation of AI-assisted metamaterial design is explainability. Neural networks and generative models may predict high performance without revealing which physical mechanism is responsible. This can be problematic in automotive engineering, where design decisions must be justified, validated, and released under safety and durability requirements [48,49,50,51].

Explainable AI can help identify whether bandgap performance is controlled mainly by beam stiffness, attached mass, damping, unit-cell spacing, host-panel coupling, or resonator placement. Feature-importance analysis, sensitivity maps, and physics-informed latent variables can make AI tools more acceptable to NVH engineers. The objective should be not only to generate a good design, but also to understand why it works and under which conditions it may fail.

Physical consistency is equally important. AI-generated designs should be checked against modal behavior, stress limits, energy conservation, boundary-condition sensitivity, and manufacturing feasibility. A design that cannot be interpreted as a plausible elastic or acoustic wave-control mechanism should not be adopted only because a surrogate model predicts a numerical performance advantage.

### 9.9. Digital Twins and Lifecycle Learning

Digital twins provide a possible long-term framework for connecting design, manufacturing, validation, and operation. A digital twin of a metamaterial-enabled EV component would combine its nominal simulation model with measured manufacturing data, beginning-of-life FRFs, environmental exposure history, and operational vibration data. This could allow the predicted bandgap location and attenuation performance to be updated throughout the component lifecycle [37,46,48].

In practical terms, a digital twin could support three functions. First, it could help qualify manufactured parts by comparing measured resonance frequencies with allowable tolerance bands. Second, it could predict whether temperature, fatigue, or aging will shift the metamaterial outside its target band. Third, it could provide feedback for future design iterations by linking real component performance to the original surrogate and FE models.

This concept is still early for vibroacoustic metamaterials, but it fits naturally with the direction of EV development. Modern vehicles and production systems generate large amounts of data, and NVH development already uses simulation–test correlation, operational measurements, and model updating. Metamaterials add a frequency-selective element to this ecosystem, making continuous learning especially valuable.

### 9.10. Limitations and Research Needs in AI-Assisted Metamaterial Design

Despite its promise, AI-assisted metamaterial design has several limitations. First, many published models are trained mainly on simulation-generated datasets. If the underlying FE model contains unrealistic damping, simplified boundary conditions, or ideal manufacturing assumptions, the AI model will reproduce these biases. Experimental data are therefore needed not only for final validation, but also for training and correcting data-driven models [33,48,49,50,51].

Second, many AI studies optimize a single performance metric, such as bandgap width or resonance frequency. Automotive implementation requires a broader objective set, including mass, stiffness, packaging, fatigue, crashworthiness, corrosion, cost, acoustic radiation, and perceived sound quality. Future studies should therefore use multi-objective and constraint-aware frameworks rather than isolated performance maximization [37,38,39,44,45,46].

Third, the connection between AI-generated metamaterial designs and vehicle-level NVH targets remains weak. Many designs are demonstrated on idealized beams, plates, or unit cells, while EV NVH problems are source–path–receiver phenomena. The next research step should be to link AI-based inverse design directly to measured or simulated EV order maps and transfer path contribution data.

AI should support physics-based design, not replace it. Its strongest contribution is the acceleration of physics-based, order-targeted, and experimentally validated metamaterial development. When coupled with robust simulation, manufacturing constraints, and vehicle-level validation, AI can help move vibroacoustic metamaterials from promising laboratory structures toward practical EV NVH components.

## 10. Sustainability and Lifecycle Aspects of Vibroacoustic Metamaterials

Sustainability matters because these treatments affect mass, materials, manufacturing energy, durability, and end-of-life handling. The acoustic problem is closely linked to mass, material use, manufacturing energy, durability, and lifecycle performance. Conventional NVH treatments often rely on added mass, multilayer absorbers, damping sheets, and auxiliary tuned absorbers. These solutions can be effective, but they may increase vehicle weight, material consumption, assembly complexity, and end-of-life separation difficulty. Metamaterials offer a different route because their attenuation is mainly achieved through architecture and local dynamic behavior rather than through bulk material addition [26,27,28,29,52,67].

This sustainability argument must be treated carefully. A metamaterial is not automatically more sustainable simply because it is lightweight or technologically advanced. Additive manufacturing may reduce material waste but can consume significant process energy. Multi-material resonator assemblies may improve acoustic performance but complicate recycling. A polymer resonator may be lightweight but may degrade under temperature, humidity, or chemical exposure. Therefore, the environmental value of a metamaterial depends on the complete lifecycle balance between acoustic benefit, mass reduction, manufacturing route, durability, repairability, and end-of-life treatment [38,44,45,46,67,68].

### 10.1. Lightweighting as an NVH and Sustainability Driver

Vehicle lightweighting is one of the strongest motivations for metamaterial-based NVH solutions. In EVs, added mass influences energy consumption, range, tire wear, brake wear, and the potential size of the battery pack. Traditional acoustic packages can be difficult to reconcile with lightweight design because their performance is often obtained by increasing surface density, layer thickness, or damping coverage. Low-frequency attenuation is especially demanding because conventional absorbers and barriers become inefficient when wavelengths are long [13,14].

Locally resonant metamaterials can improve the attenuation-to-mass ratio by concentrating dynamic functionality into small resonant structures. Instead of treating an entire panel with heavy damping material, resonators can be placed at locations where modal participation or transfer path contribution is high. This makes it possible to target the most relevant structural–acoustic paths with less added material. The wheel-arch, gearbox-housing, panel, and parcel-shelf examples reviewed earlier illustrate this principle: the goal is not to add broadband mass, but to introduce frequency-selective attenuation where it directly affects the EV noise problem [6,7,8,9,30,31,32,33].

However, the lightweighting benefit should be measured against a realistic baseline. A metamaterial solution should not be compared only with an untreated structure, but also with the best available conventional countermeasure of similar cost, mass, and packaging volume. For production engineering, the relevant question is whether the metamaterial provides more insertion loss, lower cabin tonality, or better perceived sound quality per kilogram and per unit cost than damping sheets, tuned mass dampers, acoustic foams, ribbing, or structural redesign.

### 10.2. Multifunctionality and Component Consolidation

One of the strongest sustainability opportunities is multifunctionality. Conventional automotive design often treats structural support, acoustic attenuation, crash protection, thermal insulation, and electromagnetic shielding as separate functions assigned to separate parts. Architected materials can potentially combine several of these functions in one component. For example, a battery-tray lattice may contribute to stiffness, energy absorption, vibration isolation, and packaging efficiency. A meta-structured e-drive housing may combine load-bearing stiffness with order-targeted vibration filtering. A wheel-arch liner may simultaneously provide splash protection, local acoustic shielding, and resonant tire cavity noise attenuation [7,8,31,34].

The sustainability value of multifunctionality is not limited to mass reduction. It may reduce the number of components, simplify assembly, shorten supply chains, and reduce the use of adhesives or secondary damping treatments. In addition, multifunctional designs can support circularity if they are realized as monomaterial or easily separable components. The opposite can also occur: a highly optimized multi-material metamaterial may improve NVH but become difficult to recycle. Therefore, multifunctionality should be evaluated not only by technical performance but also by lifecycle separability and manufacturing robustness. The principal sustainability opportunities, lifecycle benefits, and implementation risks associated with vibroacoustic metamaterials in electric vehicle applications are summarized in Table 14.

### 10.3. Additive Manufacturing: Sustainability Potential and Trade-Offs

Additive manufacturing has enabled many metamaterial demonstrators because it can realize complex internal lattices, folded resonators, thin beams, and multi-scale architectures that are difficult to fabricate using conventional tools. From a sustainability perspective, the relevant comparison must include feedstock preparation, build utilization, support structures, rejected parts, post-processing, transport, use-phase mass effects, and service life rather than material waste alone. The AM literature therefore cautions that design freedom and part consolidation can provide lifecycle benefits, but these benefits are process- and application-dependent rather than automatic [5,7,33,64,65,66,67].

At the same time, additive manufacturing should not be presented as an automatically sustainable solution. Metal powder-bed fusion can be energy-intensive and may require support removal, heat treatment, surface finishing, and quality inspection; polymer AM can involve anisotropy, surface roughness, aging sensitivity, and limited temperature stability. Electricity mix, machine utilization, build packing, post-processing, scrap, and component lifetime can materially change the result of a lifecycle comparison [5,7,8,33]. For high-volume EV production, injection molding, thermoforming, die casting, stamping, or hybrid manufacturing may therefore be more realistic than direct printing of every metamaterial part.

A practical industrial strategy is to use AM during concept discovery and validation, then redesign successful architectures for scalable manufacturing. For example, a metal 3D-printed wheel-arch resonator may prove the physical concept, but a production version might use a stamped metal, cast aluminum, or molded polymer geometry. Similarly, a polymer printed resonator array can be treated as a prototype for a future injection-molded monolithic component.

### 10.4. Recyclability, Material Compatibility, and End-of-Life Design

End-of-life treatment is a critical but often underdeveloped aspect of metamaterial research. Many high-performance acoustic concepts rely on combinations of stiff frames, soft damping layers, elastomers, adhesives, metallic masses, polymer membranes, foams, and coatings. These multi-material assemblies can be difficult to separate and may reduce recyclability. For vehicle-level reporting, the mass-based calculation logic of ISO 22628 provides a consistent basis for distinguishing recyclability and recoverability, although it does not replace component-specific dismantling and material-quality analysis [100].

Monomaterial metamaterials may provide a more circular design path. A polymer resonator array made from a single recyclable thermoplastic, a stamped metallic resonator panel, or an aluminum casting with integrated resonant ribs may be easier to recycle than a bonded hybrid stack. Nevertheless, monomaterial solutions may not always provide sufficient damping or temperature stability. A balanced strategy is therefore required: use multi-material systems only where they provide a clear acoustic or durability benefit, and design interfaces so that separation is possible at end of life.

For battery enclosures and e-drive housings, end-of-life design is particularly important because these components may be connected to safety-critical assemblies. Metamaterial features should not obstruct disassembly, repair, inspection, or recycling of battery modules and powertrain components. Design for disassembly should therefore be considered alongside bandgap design and structural optimization.

### 10.5. Durability as a Sustainability Requirement

A metamaterial that loses its tuning after aging is not sustainable, even if it is initially lightweight. Resonant systems are sensitive to geometry, stiffness, mass, damping, and boundary conditions. Temperature-dependent modulus changes, creep, adhesive softening, corrosion, moisture absorption, fatigue cracks, and surface damage can shift resonance frequencies and reduce attenuation. Therefore, durability is not only a reliability issue but also a sustainability issue: poor durability can lead to replacement parts, additional service interventions, or acoustic performance loss during the vehicle lifetime [38,44,45,46].

For production-ready EV metamaterials, beginning-of-life acoustic performance should be complemented by end-of-life or aged-state acoustic performance. This means that a component should be tested after thermal cycling, humidity exposure, salt spray (where relevant), vibration fatigue, impact exposure, and chemical contact with road contaminants or oils. The most important metric is not only whether the part remains mechanically intact, but whether its stop band remains aligned with the target EV order or structural resonance after aging.

### 10.6. Lifecycle Metrics for Metamaterial-Based NVH Solutions

To compare metamaterials with conventional NVH countermeasures, sustainability metrics should be linked to acoustic function. Simple mass reduction is not sufficient because an ultralight solution that provides poor attenuation may not be useful. Conversely, a slightly heavier metamaterial may be justified if it replaces several conventional components and provides strong order-specific reduction. A practical assessment should therefore combine acoustic, structural, manufacturing, and lifecycle indicators. Formal comparisons should follow the ISO 14040/14044 structure of goal-and-scope definition, lifecycle inventory, impact assessment, interpretation, and sensitivity or critical review [98,99].

For an automotive vibroacoustic metamaterial, the functional unit should express the delivered NVH service rather than only the mass of material. A suitable example is one vehicle component that maintains a specified reduction in a defined order or frequency band over an explicitly stated service life. The system boundary should then include raw-material production, manufacturing and post-processing, joining and assembly, use-phase effects of mass and replacement, maintenance, and end-of-life processing. This functional-equivalence requirement prevents a lightweight prototype from being compared unfairly with a durable production countermeasure that provides a different acoustic function [98,99]. The proposed lifecycle-oriented assessment metrics for automotive vibroacoustic metamaterials are summarized in Table 15.

### 10.7. Sustainability Assessment and Evidence Completeness

A sustainability-oriented assessment should begin with the NVH problem and a functional unit that expresses the delivered acoustic or vibration benefit. The metamaterial should first be compared with a conventional baseline at equivalent target frequency, mass, packaging, stiffness, and service conditions. Only after this functional comparison should material, process, durability, repairability, and end-of-life effects be evaluated.

**Figure 4:**
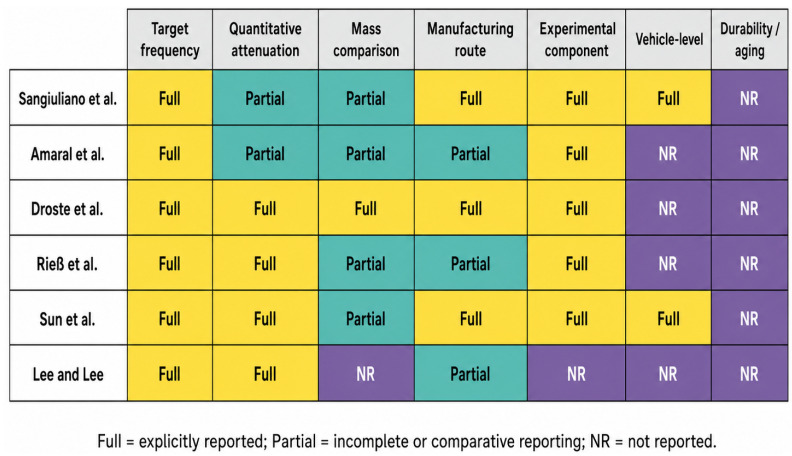
Author-generated evidence-completeness matrix for the representative automotive studies summarized in Table 4 [7,8,30,31,33,34]. Full = explicitly reported; Partial = incomplete or comparative reporting; NR = not reported. The matrix is a descriptive reporting map and not a study-quality score.

Current automotive studies report the target frequency and attenuation more consistently than durability, aging, absolute mass, and lifecycle information. Figure 4 maps this reporting completeness for the representative studies summarized in Table 4. The matrix is descriptive: it indicates the engineering evidence that was reported, but it is not a quality ranking of the studies.

### 10.8. Research Needs in Sustainability and Lifecycle Assessment

Current studies often discuss sustainability but rarely quantify it across the full lifecycle. Many papers report mass savings or compactness, but fewer define a functional unit, system boundary, inventory dataset, impact categories, or uncertainty analysis in accordance with established LCA practice [98,99]. Similarly, durability is often acknowledged as a challenge, but long-term post-aging acoustic measurements remain scarce. This creates a gap between promising laboratory demonstrations and credible industrial sustainability claims.

Future research should therefore combine vibroacoustic performance testing with lifecycle assessment, production-feasibility studies, and durability validation. Particularly useful studies would compare a metamaterial solution with a conventional damping or dynamic-absorber baseline using the same vehicle-level target, acoustic metric, service life, mass-accounting convention, and end-of-life scenario. Process-energy and material-circularity claims should be supported by transparent inventory assumptions and sensitivity analysis rather than by generic statements that lightweighting or additive manufacturing is inherently sustainable [97,98,99,100].

Another important direction is the development of recyclable and repairable metamaterial architectures. Monomaterial polymer resonators, stamped metallic meta-panels, detachable resonator modules, and integrated cast structures may provide more realistic industrial pathways than fragile or inseparable multi-material laboratory assemblies. Digital twins and data-driven aging models may also support lifecycle monitoring by predicting resonance drift, fatigue risk, and residual acoustic performance during vehicle operation [36,37,47,48,49,50,51,52,53].

## 11. Technology Readiness and Industrial Adoption Roadmap

Vehicle-relevant studies now cover wheel arches, housings, panels, doors, covers, and battery structures, but maturity varies widely. Readiness must therefore be assessed component by component according to durability, manufacturability, cost, structural responsibility, and vehicle-level validation [6,7,8,9,30,31,32,33,34,35].

### 11.1. Technology Readiness Assessment Principles

The review uses a conservative engineering-readiness scale: theory and unit-cell simulation are early-stage; coupon, plate, and rig tests are intermediate; and realistic component or vehicle demonstrations are more mature. Assessment also requires predictable physics, tuning to a real EV source, scalable manufacture, environmental stability, and receiver-level benefit [38,44,45,46].

### 11.2. Current Maturity by Application Area

Among the reviewed application areas, wheel-arch resonators and selected body-panel or cover-integrated solutions appear closest to near-term implementation. They are attractive because they can be attached to or integrated into components that are not always primary load-bearing structures. Their acoustic target can also be relatively well defined, such as tire cavity resonance or a cover-panel tone. The wheel-arch study using metal 3D-printed resonant metamaterials is particularly important because it demonstrates a vehicle-relevant path rather than only a generic laboratory specimen [7].

Gearbox and e-drive housings offer high value because they address radiation near the source–transfer interface, but they require preservation of alignment, sealing, lubrication, thermal behavior, casting feasibility, fatigue strength, and modal robustness [8]. Battery meta-structures remain less mature because crash, fire, electrical safety, sealing, durability, and serviceability dominate the validation burden [34,35]. The technology-readiness-oriented assessment of the principal EV vibroacoustic metamaterial application areas is summarized in Table 16.

### 11.3. Industrial Barriers Beyond Acoustic Performance

Industrial barriers arise from non-ideal boundaries, joints, curvature, thermal gradients, manufacturing scatter, detuning, fatigue, and debonding. Prototype AM routes must also be converted to molding, forming, casting, or hybrid assembly where high-volume cost and repeatability demand it [38,44,45,46].

A further barrier is responsibility within the vehicle development process. Metamaterials sit between materials engineering, structural dynamics, acoustics, CAE, manufacturing, durability, and cost engineering. If no single engineering group owns the complete source–path–receiver performance of the meta-structured component, promising concepts may remain in research rather than moving into platform development. Industrial adoption therefore requires process integration as much as technical maturity.

### 11.4. Short-Term, Mid-Term, and Long-Term Adoption Scenarios

A staged roadmap is most realistic. Short-term applications are add-on or replaceable resonators for wheel arches, covers, trim, and panels; mid-term systems integrate resonators into molded, stamped, cast, or lattice-reinforced components during design rather than as late corrections [6,7,8,9,30,31,32,33].

In the long term, the most advanced scenario is multifunctional and adaptive metamaterial integration. Battery trays, structural enclosures, and body panels may be designed as architected systems that combine stiffness, crash energy absorption, vibration filtering, thermal management, and acoustic radiation control. Active or tunable metamaterials may also become relevant if they can track speed-dependent motor and gear orders with acceptable reliability, power consumption, cost, and fail-safe behavior [36,37,47,48,49,50,51,52,53]. The proposed industrial adoption roadmap, from concept screening and component validation to production-intent development and vehicle-level implementation, is summarized in Table 17.

### 11.5. Integration into Automotive Development Workflows

Production development should begin with a measured or simulated EV problem and transfer path ranking, followed by component selection and only then unit-cell or resonator design. Each study should compare the metamaterial with a conventional baseline such as a damper, liner, rib, wall-thickness change, foam, or material substitution.

### 11.6. Recommended Minimum Evidence Package for Production-Oriented Studies

A minimum evidence package should report the target problem and order, baseline, component and added mass, predicted bandgap, measured insertion loss, boundary conditions, materials, manufacturing route, tolerance sensitivity, durability status, and—where relevant—cabin SPL, sound power, or psychoacoustic benefit [1,2,3,4,40,41,42,43]. Limitations such as numerical-only validation, flat-plate testing, or AM scalability should be stated explicitly.

### 11.7. Synthesis: Most Promising Adoption Path

The most credible near-term path is a passive, robust, production-compatible resonator or meta-panel for one well-defined tone, especially in wheel arches, covers, or selected panels. Longer-term value lies in multifunctional housings and enclosures that preserve stiffness, mass efficiency, manufacturability, durability, and lifecycle stability.

## 12. Research Gaps and Future Directions

VAMMs have progressed from theoretical wave-control concepts to automotive demonstrators, but broad EV deployment is limited by translation into robust, scalable, lifecycle-validated components. The following gaps focus on order-targeted design, vehicle validation, durability, manufacturing, benchmarking, AI reliability, and multifunctional development [6,7,8,9,20,23,26,27,30,31,32,33,34].

### 12.1. Gap 1: Limited EV-Specific and Vehicle-Level Validation

Most studies still use idealized unit cells, beams, plates, or coupons whose stop bands may shift or weaken in curved, jointed, damped, thermally loaded vehicle structures. Component-oriented wheel-arch, housing, door, cover, and battery studies are valuable, but full-vehicle A/B tests remain rare; future work should begin from real EV problems and close the validation chain at component or vehicle level [1,2,3,4,6,7,8,9,29,30,31,32,33,34,35].

### 12.2. Gap 2: Weak Connection Between EV Order Analysis and Bandgap Tuning

Many studies target a fixed frequency, although gear, motor, inverter, and bearing orders sweep with speed and load. More relevant designs should derive bandgaps and resonator placement from order maps, ODS, transfer path analysis, and receiver measurements so that attenuation overlaps the dominant radiating path [1,6,7,8,10,11,12,13].

### 12.3. Gap 3: Insufficient Durability, Aging and Environmental Robustness Data

Resonators can experience cyclic stress, fatigue, creep, moisture, thermal aging, stiffness degradation, debonding, and rattle, all of which detune performance. Studies should therefore report beginning- and end-of-life response after thermal cycling, humidity, salt spray (where relevant), random vibration, impact, and attachment tests [24,38,39,44,45,46].

### 12.4. Gap 4: Manufacturing Scalability and Quality Control

AM enables complex demonstrators, but high-volume vehicles usually require molding, forming, casting, extrusion, or composite processes. Production-oriented studies should report feasible routes, tolerances, property scatter, resonance-frequency variability, and frequency-response-based quality-control criteria [7,8,33,34].

### 12.5. Gap 5: Lack of Standardized Benchmarking Metrics

Cross-study comparison is hindered by inconsistent metrics and baselines. At a minimum, publications should report target band, conventional baseline, added mass, attenuation in dB, validation level, order and operating condition, temperature, and vehicle-level effect; psychoacoustic metrics are needed for perceptually tonal problems [1,2,3,4,6,7,32,40,41,42,43,44]. The main research gaps and the corresponding recommended actions for advancing EV vibroacoustic metamaterial development are summarized in Table 18.

### 12.6. Gap 6: AI-Assisted Design Still Requires Physical Constraints

AI, surrogate modeling, topology optimization, and inverse design are useful only when solutions remain physically interpretable, manufacturable, robust, and experimentally validated. Objectives should extend beyond bandgap frequency to mass, stress, fatigue, stiffness, tolerance and temperature sensitivity, and component-level insertion loss [36,37,47,48,49,50,51,52,53].

### 12.7. Gap 7: Limited Integration with Multifunctional and Sustainable Vehicle Design

Long-term value depends on multifunctionality: battery enclosures, housing ribs, or liners should combine NVH with structural, crash, thermal, protective, and lifecycle functions. Optimization must therefore include stiffness, strength, crashworthiness, recyclability, repairability, and manufacturing energy [8,34,55,64,65,66,67,68].

### 12.8. Future Direction 1: Order-Targeted Meta-Structured E-Drive Housings

Order-targeted e-drive housings are a high-value direction: multibody, electromagnetic, structural, and acoustic models can identify critical orders and radiating panels, after which resonators or tuned ribs are placed to reduce wall velocity, sound power, cabin SPL, and mass-normalized response [6,7,8,10,11,12,13].

### 12.9. Future Direction 2: Metamaterial Battery Enclosures and Multifunctional Trays

Battery enclosures represent a second strategic future direction. Large tray and cover structures are exposed to road-induced vibration, structural modes and potential acoustic radiation. Lattice, auxetic and hybrid meta-core designs may reduce vibration transmission while also contributing to stiffness and crash energy absorption [34,35,55,64,65,66,67,68,69,70]. However, this application requires particularly careful validation because battery enclosures are safety-critical structures. Any vibroacoustic improvement must be evaluated together with crashworthiness, sealing, corrosion resistance, thermal management and repairability.

### 12.10. Future Direction 3: Robust Low-Cost Metamaterial Patches and Liners

Near-term research should prioritize low-cost molded liners, trim-integrated arrays, cover features, and bonded patches that outperform conventional treatments at equal or lower mass, cost, assembly complexity, rattle risk, and tuning sensitivity [6,7,9,30,31,32,33].

### 12.11. Future Direction 4: Adaptive and Tunable Metamaterials for Speed-Dependent Orders

Adaptive systems may eventually follow speed-dependent orders using piezoelectric shunts, variable-stiffness or magnetorheological elements, and controlled resonator arrays, but only if reliability, fail-safe behavior, temperature robustness, electrical integration, and vehicle-level sound-quality benefit justify the added complexity [36,37,47,48,49,50,51,52,53].

### 12.12. Integrated Future Research Priorities

The priorities summarized in Table 19 should be treated as an integrated development sequence rather than as isolated research topics. Future studies should begin from a specific EV NVH source and transfer path, identify a stable target order or frequency band, select a component-compatible mechanism, include manufacturing and durability constraints during optimization, and finish with component- and vehicle-level evidence. This sequence reduces the risk of producing physically elegant but industrially disconnected demonstrators.

### 12.13. Critical Synthesis and Engineering Decision Framework

The evidence supports a differentiated deployment strategy. Passive local and hybrid resonators have the strongest near-term basis for wheel arches, panels, covers, and housing-related structures; lattice and auxetic systems are promising for multifunctional battery enclosures but remain mainly numerical; membrane and cavity concepts suit compact covers and trim; and active, topological, and pentamode systems are longer-term options [6,7,8,9,12,13,17,19,20,21,24,25,30,31,32,33,34,35,36,37,47,48,49,50,51,52,53]. The evidence-based engineering selection summary for the principal EV vibroacoustic metamaterial concepts is presented in Table 20.

The practical decision rule is therefore source- and path-specific. A metamaterial is most justified when the disturbance is tonal or order-related, the treatment location is identifiable, and mass or packaging constraints make conventional measures inefficient. Conventional damping, isolation, absorption, or structural modification should remain the preferred solution for broadband, uncertain, low-cost, or safety-critical cases; hybrid solutions are often the most realistic choice when a narrowband tone is superimposed on a broadband background.

## 13. Conclusions

This review evaluated vibroacoustic metamaterials as engineering tools for low-frequency electric vehicle NVH control. In this context, low frequency denotes the application-specific range below 1000 Hz, with primary emphasis on approximately 50–1000 Hz. The principal opportunity is not universal sound absorption, but mass-efficient attenuation of identifiable tonal or order-related sources through geometry-driven control of structural and acoustic wave propagation.

The strongest near-term evidence supports passive locally resonant and hybrid multi-resonant concepts for wheel arches, covers, doors, selected body panels, and gearbox-related structures. Lattice and auxetic systems offer greater multifunctional potential for battery enclosures and large lightweight structures but remain more dependent on numerical evidence and require crash, thermal, sealing, and durability validation. Active, tunable, topological, and pentamode systems expand the future design space but are not yet supported by comparable automotive maturity.

A production-relevant development process should begin with the EV source–path–receiver problem rather than an isolated unit cell. Critical orders and transfer paths should be identified first; the metamaterial should then be tuned under mass, stiffness, manufacturing, temperature, and tolerance constraints. Evidence should progress from band-structure and finite-component analysis to component, environmental, subsystem, and vehicle-level validation using directly comparable acoustic and vibration metrics.

Industrial feasibility will depend on converting laboratory geometries into repeatable stamping, molding, casting, thermoforming, composite, or hybrid production routes. Additive manufacturing remains valuable for rapid design exploration but does not by itself demonstrate series-production suitability. Sustainability claims must likewise be based on functional equivalence, mass-normalized attenuation, manufacturing energy, service life, repairability, and end-of-life performance rather than lightweight geometry alone. Physics-aware AI and inverse design can accelerate this process only when these engineering constraints and experimental feedback are included.

Overall, vibroacoustic metamaterials are best viewed as selective additions to the established NVH toolbox. Their short-term role is targeted passive control in components with stable tonal problems and manageable structural responsibility. Their medium-term potential lies in order-targeted e-drive housings and multifunctional enclosures. Progress toward production requires standardized reporting of target frequency, attenuation metric, baseline, added mass, environmental stability, manufacturing variability, cost, and vehicle-level benefit.

## Figures and Tables

**Table 1:** Critical comparison of vibroacoustic metamaterial families for electric vehicle NVH applications.

Mechanism/Family	Dominant Physical Principle	Best Operating Regime and Key Advantage	Best-Fit EV Sources or Components	Principal Disadvantage/Boundary of Use	Representative References
Phononic crystal/Bragg scattering	Periodic impedance contrast and destructive interference	Clear wave-filtering bands at mid-to-high frequencies where the lattice spacing is feasible	Periodic ribs, structured panels, higher-frequency housing or body-panel wave filtering	Unit-cell dimensions become impractical for most low-frequency vehicle targets below 1 kHz	[16,17,18,19,29,56,57,58,59]
Locally resonant metamaterial	Out-of-phase internal resonator motion and frequency-dependent effective inertia or stiffness	Compact subwavelength stop bands and high attenuation around a fixed tonal target	Gearbox housings, wheel arches, covers, doors, body panels, and local transfer paths	Narrowband behavior and detuning caused by temperature, aging, attachment stiffness, and manufacturing scatter	[6,7,8,20,21,22,23,24,25,26]
Hybrid/multi-resonant meta-structure	Overlapping local resonances or combined local-resonance, Bragg, membrane, or cavity mechanisms	Several nearby tones or a wider targeted band than a single-resonator design	Gear mesh harmonics, motor-order clusters, power-electronics covers, and multi-modal panels	Higher mass and design complexity; inter-resonator coupling can create amplification outside the intended stop band	[24,25,26,29,36,37,38]
Membrane-type metamaterial	Membrane tension coupled with attached or distributed inertia	Very thin low-frequency sound-transmission-loss treatment	Interior trim, lightweight covers, liners, and secondary acoustic packages	Pre-tension, polymer aging, temperature sensitivity, fire requirements, and limited load-bearing capability	[23,26]
Helmholtz/labyrinthine/coiled-space concept	Air mass and cavity-compliance resonance or a compact folded acoustic path	Low-frequency airborne attenuation in a shallow package	HVAC or compressor packages, covers, cavities, ducts, and local airborne paths	Narrowband tuning, contamination, airflow losses, moisture, and packaging constraints	[14,26,27,28]
Lattice, auxetic, and pentamode mechanical metamaterial	Architecture-controlled effective stiffness, density, deformation mode, and energy distribution	Multifunctional vibration isolation, stiffness tailoring, energy absorption, and load-path integration	Battery trays and covers, floor structures, protective panels, supports, and sandwich cores	Crash, fatigue, thermal, sealing, electrical-safety, and repairability evidence remains limited	[34,35,55,64,65,66,67,68,69,70]
Topological acoustic/elastic metamaterial	Band-topology-controlled interface or edge-state waveguiding	Potentially defect-tolerant routing of vibration away from sensitive radiating regions	Future body structures, protected wave paths, and robust interfaces	Low automotive maturity, complex geometry, and limited evidence under realistic damping and finite boundaries	[27,29,59]
Active or tunable metamaterial	Adaptive stiffness, damping, shunt impedance, or controlled feedback	Can follow moving motor or gear orders that exceed the bandwidth of a passive stop band	Future smart e-drive covers, adaptive panels, mounts, and reconfigurable resonator arrays	Sensors, controllers, power, EMC, fail-safe behavior, diagnostics, cost, and lifetime reliability	[36,37,49]

Note. The table is a mechanism-selection aid rather than a universal ranking. The preferred family depends on the source, transfer path, target-frequency stability, structural function, environment, and acceptable manufacturing complexity.

**Table 2:** Mechanism-to-application decision matrix for low-frequency electric vehicle NVH control.

EV NVH Source/Problem	Dominant Physics and Transfer Path	Preferred Metamaterial Concept	Why It Is the Best-Fit Concept	Selection Boundary/When Another Solution May Be Preferable	Representative References
Motor orders and electromagnetic excitation	Speed-dependent radial-force and torque harmonics transmitted through the stator, frame, bearings, and housing	Graded or multi-resonant structural resonators; tunable systems for wide order sweeps	Can span several nearby orders or adapt when a fixed passive band would be too narrow	A fixed passive LRM is appropriate only for a narrow critical speed window. Conventional isolation or active control may be preferable for very wide sweeps.	[1,6,13,31,36,52]
Inverter and power-electronics tonal noise	Switching-related tones, sidebands, and vibration of covers, shields, and mounts	Cover-integrated locally resonant, membrane, or hybrid acoustic structural treatment	Targets the radiating cover directly within a compact package and can be integrated without modifying the electronic source	Constrained-layer damping, encapsulation, or conventional shielding may be superior for broadband high-frequency content or where thermal and EMC margins are critical.	[1,13,31,46]
Gear whine and housing radiation	Transmission error and mesh-stiffness harmonics transmitted through shafts and bearings to radiating housing walls	Order-targeted locally resonant ribs, inserts, or housing-wall meta-structures	The source–path–receiver chain is well defined and the dominant tonal orders can be matched to a passive stop band	Stiffness optimization, ribbing, or damping remains preferable where the excitation is broadband or bearing alignment, lubrication, and durability dominate.	[8,10,11,13]
Tire cavity resonance and road-noise transfer	Relatively stable 180–250 Hz cavity resonance coupled through the wheel, suspension, wheel arch, and body	Wheel-arch or liner-integrated locally resonant arrays	A compact resonator can target a well-defined low-frequency path and avoid a bulky concentrated damper	Conventional cavity absorbers or dynamic dampers may remain preferable when cost, contamination, impact resistance, corrosion, or attachment durability dominate.	[6,7,13]
Auxiliary compressor, HVAC, pumps, and actuators	Component-specific tonal forces, cover radiation, bracket vibration, and local airborne paths	Membrane, Helmholtz, or compact local-resonator cover or mount treatment	Small resonant packages can be tuned to an identifiable tonal source in a restricted space	Conventional mufflers, absorbers, isolation mounts, or airflow treatments are better for broadband flow noise or where contamination and service access are critical.	[1,6,13,44,45,46]
Battery enclosure vibration	Road-induced random input and low-order modes of large trays, covers, and floor-connected structures	Lattice, auxetic, pentamode, or multifunctional sandwich meta-core	These architectures can combine vibration control with stiffness, energy absorption, and structural integration	Conventional stiffening or sandwich cores remain preferable until crash, thermal, sealing, electrical-safety, fatigue, and repairability requirements are demonstrated.	[34,35,67,68]
Body-panel booming, doors, and trim radiation	Narrow panel modes excited by road, drivetrain, actuators, or local structure-borne paths	Distributed resonant patches, stamped resonator patterns, or trim-integrated arrays	Targets high-contribution modes while avoiding full-surface damping-sheet mass	Constrained-layer damping is usually better for broadband response or high modal density; resonant treatments require stable boundaries and attachment stiffness.	[6,9,13,30,31,32,33]
Psychoacoustically dominant tonal annoyance	Receiver-level tonality, sharpness, roughness, or order prominence rather than the largest structural amplitude alone	Psychoacoustically weighted passive, hybrid, or tunable metamaterial optimization	Directs attenuation toward the frequencies that most strongly affect perceived sound quality	Broadband acoustic packages or active noise control may be more effective when annoyance is not associated with a stable tonal or path-specific component.	[1,2,3,4,40,41]

Note. This matrix identifies the preferred concept for each source–path problem but does not imply that metamaterials are always superior. Conventional damping, absorption, stiffening, isolation, or active noise control should be retained when they offer a broader, simpler, more durable, or more cost-effective solution.

**Table 3:** Representative automotive applications of vibroacoustic metamaterials.

Application Area	Target NVH Problem	Metamaterial Concept	Validation Level	Main Reported Relevance	Key Limitation	References
Gearbox/e-drive housing	Gear whine, bearing-force transfer, and housing radiation	Locally resonant metamaterial integrated into a lightweight metallic housing or rib architecture	Numerical and experimental gearbox-housing validation	Reduces radiated sound power in predicted bandgap regions while supporting lightweight housing design	Must preserve stiffness, bearing alignment, thermal behavior, lubrication, sealing, durability, and manufacturability	[8,10,11,13]
Electric motor casing/stator support	Electromagnetic radial forces, motor orders, and housing vibration	Order-targeted locally resonant or tunable casing treatment; possible piezoelectric or adaptive meta-shell	Mostly conceptual or transferable from smart-structure studies	Potential to attenuate tonal orders before they radiate through the motor or e-drive enclosure	Excitation frequencies vary with speed; thermal, EMC, controller reliability, and packaging constraints are severe	[1,6,31,36,37,49]
Rear wheel arches	Structure-borne tire cavity noise around 180–250 Hz, commonly near 230 Hz	Metal 3D-printed resonant elements attached to wheel-arch structures	Vehicle-level experimental verification	Demonstrates compact resonant metamaterials as an alternative to conventional dynamic damper solutions	Metal additive manufacturing (AM) cost, corrosion protection, attachment durability, contamination, and tolerance robustness remain production challenges	[6,7,13]
Battery pack casing/tray/cover	Road-induced random vibration, enclosure panel modes, and possible floor radiation	Lattice, auxetic, and cellular mechanical metamaterial structures integrated into casing or core regions	Mainly numerical battery-casing studies	Shows strong vibration mitigation potential, especially for aluminum casings, while opening a multifunctional structural route	Crashworthiness, thermal management, sealing, fatigue, electrical safety, and aging need much stronger validation	[34,35,64,65,67,68]
Vehicle panels/doors/dash structures	Local panel radiation, door-panel vibration, dash-panel transmission, and tonal structure-borne noise	Distributed resonant patches, stamped resonant patterns, or integrated resonator arrays	Component or vehicle demonstrator level in selected studies	Useful for targeted panel velocity reduction without the mass penalty of broad damping-sheet coverage	Sensitive to attachment stiffness, local curvature, manufacturing scatter, crash packaging, corrosion, and frequency detuning	[6,9,30,32,33]
Power-electronics covers	Cover vibration excited by inverter, switching-related tones, coolant pump excitations, and e-drive structure-borne paths	Cover-integrated vibroacoustic metamaterial or locally resonant cover treatment	Component-level or demonstrator evidence	Attractive because covers are easier to redesign than load-bearing housings and can target tonal radiation directly	Must remain compatible with sealing, thermal management, electromagnetic compatibility, serviceability, and cost	[6,31,46,49]
Auxiliary compressor, HVAC, pumps, and actuators	Low-frequency hum, tonal compressor orders, and local bracket or cover vibration	Compact membrane, Helmholtz, or local-resonator treatments integrated into covers, mounts, or surrounding acoustic packages	Mostly transferable concept and early-stage application evidence	Suitable for small tonal sources where conventional bulky treatments are difficult to package	Narrowband tuning, airflow, contamination, thermal aging, and service access must be considered	[1,6,14,26,27]
Interior trim and secondary acoustic packages	Cabin-side tonal radiation, trim-panel vibration, and local acoustic transmission	Membrane-type, labyrinthine, coiled-space, or polymeric resonant acoustic metamaterial panels	Laboratory to component-level evidence	Can improve low-frequency attenuation in thin packages compared with conventional porous absorbers alone	Performance may be strongly affected by mounting, pre-tension, humidity, fire requirements, recyclability, and cost	[9,14,23,26,27]
Future active EV structures	Speed-dependent motor orders, gear orders, and moving tonal components during acceleration or regenerative braking	Tunable resonators, piezoelectric shunts, adaptive meta-structures, or optimized meta-shells	Mostly laboratory, simulation, or conceptual stage	Potential to track changing order frequencies rather than suppressing only one fixed band	Requires sensors, control electronics, fail-safe design, EMC validation, lifecycle reliability, and robust calibration	[36,37,38,49]

Note. Validation levels are interpreted from the reported study context rather than treated as formally assigned technology readiness level (TRL) values.

**Table 4:** Quantitative comparison of representative automotive and EV vibroacoustic metamaterial studies.

Study/Component	Target Frequency	Reported Attenuation	Metric and Baseline	Added Mass/Mass Comparison	Performance-to-Mass	Validation Level	Ref.
Sangiuliano et al. Rear wheel arches	≈230 Hz tire cavity resonance	Vehicle-level reduction demonstrated; exact dB value NR in the extractable summary	Interior structure-borne noise and wheel-arch response; untreated/conventional damper context	Lightweight metal resonators; total added mass NR	NR	Numerical and vehicle-level experimental	[7]
Amaral et al. Gearbox housing	Predicted locally resonant bandgaps within the investigated spectrum	Significant sound-power and vibration reduction within the predicted bandgaps; exact dB varies by configuration	Radiated sound power and housing vibration; topology-optimized lightweight housing baseline	Lightweight passive concept; exact added mass NR	NR	Numerical and experimental component validation	[8]
Droste et al. Vehicle-door demonstrators	450–600 Hz design band; measured stop band ≈ 300–800 Hz	>10 dB simulated reduction; 20 dB measured peak reduction at 500 Hz	Averaged acceleration FRF relative to the reference door/curved plate	154 × 2.5 g = 0.385 kg in the door-level numerical configuration	≈52 dB kg^−1^ at the measured peak (screening indicator only)	Validated FE model and experimental curved-plate demonstrators	[30]
Rieß et al. Power-electronics cover	Integrated stop band ≈ 400–600 Hz; 400–1500 Hz design range	>30 dB structural-vibration reduction and >10 dB radiated sound-power reduction	Structural response and radiated sound power relative to a 2 mm reference cover and conventional treatments	Integrated concept adds no discrete resonator mass; exact configuration masses reported comparatively, not in kg	Not calculable from reported mass data	Numerical and experimental component validation	[31]
Sun et al. Parcel-shelf/body-boom treatment	First-order resonance below 50 Hz (≈37.5 Hz target)	>9 dB vehicle attenuation; >7 dB better than an equal-mass asphalt damper	Vehicle response at the first parcel-shelf resonance; untreated and equal-mass asphalt baselines	Mass matched to the asphalt comparator; absolute mass NR	NR	Experimental vehicle validation	[33]
Lee and Lee Battery pack casing	Random-vibration regime; low casing modes shifted above 200 Hz in aluminum designs	≈97–99% vibration reduction for aluminum casings; location-dependent 63.8% and 92.8% examples for PPA-CF	Acceleration/vibration response relative to conventional aluminum or PPA-CF casings	Relative-density and added-mass data NR	NR	Numerical only	[34]

**Table 5:** Material systems for automotive vibroacoustic metamaterials.

Material System	Key Advantages	Main Limitations	Typical Manufacturing Routes	Representative EV/NVH Relevance	References
Aluminum alloys	Low density, corrosion resistance, castability, AM compatibility, suitable for structural housings.	Low intrinsic damping; fatigue and local stress concentration must be controlled.	Die casting, machining, metal AM, hybrid casting/printing.	Gearbox housings, e-drive casings, battery enclosures, structural covers.	[8,34]
Steel alloys	High stiffness, good fatigue resistance, mature automotive forming infrastructure.	Higher density than aluminum or polymers; added mass can reduce lightweighting benefit.	Sheet-metal forming, stamping, welding, joining.	Door panels, body reinforcements, stamped resonator patterns, brackets.	[6,9,30]
Polymeric materials	Low mass, low cost, intrinsic damping, good compatibility with molding and trim integration.	Temperature sensitivity, creep, moisture absorption, aging, possible detuning.	Injection molding, thermoforming, extrusion, polymer AM.	Wheel-arch liners, covers, acoustic panels, trim-integrated resonators.	[7,31,32,33]
Fiber-reinforced composites	High specific stiffness, lightweight structural integration, design flexibility.	Joining complexity, anisotropy, recyclability issues, uncertain resonator–host interaction.	resin transfer molding (RTM), compression molding, layup, hybrid molding.	Battery covers, underbody panels, lightweight body structures.	[34,67]
Lattice and cellular architectures	Strong multifunctionality; tunable stiffness, energy absorption, and vibration transmission.	Manufacturing complexity; quality control and fatigue validation are difficult.	Metal/polymer AM, insert molding, hybrid manufacturing.	Battery trays, structural enclosures, supports, crash-energy absorbers.	[34,35,64,65,66,67,68]
Auxetic materials	Negative Poisson ratio, energy absorption, indentation resistance, tunable dynamic response.	Low production maturity; crash and durability validation still limited.	AM, advanced molding, cellular foam processing.	Battery protection, vibration isolation, multifunctional lightweight structures.	[34,35,55,69,70]
Hybrid multi-material systems	Can combine structural stiffness, damping, acoustic absorption, and resonant attenuation.	Interface durability, recyclability, manufacturing cost, and repairability are challenging.	Adhesive bonding, overmolding, co-curing, hybrid assembly.	Advanced panels, covers, sandwich structures, multi-path NVH treatments.	[6,30,31,38]

**Table 6:** Evidence-supported comparison of manufacturing routes for vibroacoustic metamaterials in electric vehicle applications.

Route	Suitable VAMM	Advantage	Main Risk	Scalability	Applications	Refs.
Metal AM	Metal resonators, lattice inserts, and cantilevers.	Excellent geometric freedom and rapid prototype iteration.	High cost, slow throughput, surface quality, residual stress, and fatigue uncertainty.	Low to medium; mainly prototypes, premium, or low-volume parts.	Wheel-arch resonators, inserts, and prototype e-drive features.	[7,8,39]
Polymer AM	Monolithic resonator arrays, trim prototypes, and acoustic lattices.	Low-cost prototyping and complex geometry realization.	Material aging, dimensional scatter, and limited production rate for series vehicles.	Medium for prototypes; limited for high-volume production.	Interior panels, covers, and low-frequency resonator arrays.	[33,38,55,67]
Injection molding	Polymer resonator arrays, covers, liners, and trim-integrated VAMMs.	Low unit cost and repeatable quality at high volume.	High tooling cost; geometry must be moldable; shrinkage and property scatter can affect tuning.	High when the architecture is redesigned for the molding process.	Wheel-arch liners, covers, and interior acoustic parts.	[30,31,32,33,38,55,67]
Thermoform	Large structured polymer panels and liners.	Suitable for large lightweight parts with moderate feature complexity.	Limited undercuts and lower geometric precision than molding or AM.	High for production-compatible shallow geometries.	Undercovers, wheel-arch liners, and trim panels.	[6,9,32,55,67]
Sheet-metal forming	Stamped resonator patterns, ribbed panels, and metallic inserts.	Very high production maturity and low unit cost.	Limited geometric freedom; springback and forming tolerances may detune features.	Very high for geometries compatible with forming limits.	Door panels, dash structures, brackets, and body-panel features.	[9,30,31,32,38]
Casting	Integrated metallic housing features and ribs.	Suitable for aluminum housings and structural integration.	Complex features can affect mold filling, porosity, machining, dimensional stability, and fatigue.	High if the geometry is production-adapted and validated.	Gearbox housings, e-drive casings, and battery frames.	[8,39,55,64,65,66,67,68]
Composite processing	Sandwich panels and composite skins with cores or resonators.	High specific stiffness and multifunctional integration.	Joining, repair, anisotropy, cost, and recycling challenges.	Medium and strongly component-dependent.	Battery covers, underbody panels, and lightweight body structures.	[34,35,55,67,68]
Hybrid assembly	Multi-material VAMMs combining resonators, damping layers, and hosts.	Allows functional separation and tuning flexibility.	Interface durability, assembly cost, rattling, and quality-control risks.	Medium for modular designs; lower for complex assemblies.	Advanced covers, body-panel treatments, and retrofit-style NVH solutions.	[6,30,31,38,55,67]

Note. The scalability assessments are qualitative author syntheses based on the cited literature, demonstrated component geometries, and compatibility with established automotive processes; they are not claims of validated production-volume implementation. Additively manufactured feasibility must be revalidated after conversion to production-intent materials and processes.

**Table 7:** Evidence-supported production risks and mitigation strategies for automotive vibroacoustic metamaterials.

Risk Category	Typical Failure or Uncertainty Mode	Potential NVH Consequence	Mitigation Strategy	Evidence/Standards
Thermal detuning	Temperature-dependent stiffness of polymers, adhesives, membranes, or elastomers.	The stop band shifts away from the target gear, motor, tire, or panel frequency.	Use temperature-dependent material data; design wider or multi-resonant bands; validate across the service-temperature range.	[38,46]
Fatigue of resonators	Cracking of slender beams, lattice struts, bonded masses, or AM features under cyclic loading.	Loss of attenuation, structural failure, or secondary noise.	Use fatigue-aware topology, reduce stress concentration at roots, apply conservative strain limits, and perform durability testing.	[39,44,45,46]
Attachment degradation	Adhesive aging, partial debonding, fastener looseness, or interface creep.	Reduced boundary stiffness, detuning, and rattling noise.	Prefer integrated or monolithic designs where possible; qualify bonding systems and repeat FRF testing after aging.	[30,31,38,46]
Manufacturing scatter	Variation in beam thickness, resonator mass, cavity volume, attachment stiffness, or lattice geometry.	Bandgap variability across components and production lots.	Use tolerance-aware optimization, robust band design, critical-dimension control, and sample-based dynamic quality checks.	[38,42,43,44,45]
Corrosion and contamination	Road salt, water, oil, debris, or surface degradation affecting exposed resonators and joints.	Changed damping or stiffness, reduced durability, and noise from loose or damaged parts.	Use protective coatings, sealed or shielded layouts, compatible materials, and environment-specific testing.	[46]
Recyclability and repair	Bonded multi-material systems or embedded resonators that are difficult to separate, inspect, or replace.	Lifecycle and serviceability drawbacks despite an initial NVH benefit.	Prefer monomaterial or separable architectures and include repairability and end-of-life criteria in concept selection.	[55,67,68]

Note. The risk categories are supported by the cited metamaterial literature and vehicle environmental or material-test standards. The mitigation strategies are engineering recommendations synthesized by the author and should be validated for the specific component, material system, and duty cycle.

**Table 8:** Evidence-supported modeling and simulation methods for vibroacoustic metamaterials in electric vehicle NVH applications.

Method	Primary Purpose	Typical EV Application	Main Advantage	Main Limitation	Refs.
Unit-cell Bloch–Floquet analysis	Prediction of dispersion curves and ideal bandgaps.	Early design of periodic resonator arrays, phononic cells, and lattice structures.	Efficient and physically transparent.	Assumes ideal periodicity and does not prove finite-component or vehicle performance.	[16,17,18,19,20,21,22,29,56,57,58,59]
Lumped-parameter resonator modeling	Rapid estimation of resonance frequency and tuning trends.	Initial design of beam, mass-spring, membrane, and Helmholtz resonators.	Fast, interpretable, and useful in the concept phase.	Cannot capture complex geometry, finite-array effects, interface compliance, or acoustic radiation.	[20,21,22,23,24,25,26]
Component-level FEM	Prediction of modal response, FRFs, insertion loss, stress, and mobility.	Gearbox housing, wheel arch, battery tray, door, cover, and body panel.	High geometric and material fidelity.	Computationally expensive for detailed repeated resonators and sensitive to damping data.	[8,13,30,31,32,33,34]
Harmonic response analysis	Evaluation of tonal or order-related response over a frequency sweep.	Gear whine, motor orders, inverter tones, compressor tones, and panel resonances.	Directly relevant for narrowband EV tonal NVH.	Sensitive to damping, boundary-condition, and excitation assumptions.	[6,7,8,31,33]
Random vibration analysis	Prediction of broadband road-induced vibration and RMS or stress response.	Battery enclosures, underbody panels, and floor structures.	Useful for road input and durability-related vibration.	Less direct for narrow resonance tuning and psychoacoustic tonal effects.	[34,35]
FEM-BEM structural–acoustic coupling	Prediction of radiated sound power, SPL, and radiation efficiency.	E-drive housings, covers, body panels, and enclosures.	Connects structural response to acoustic output.	High computational cost and dependence on accurate structural surface velocities.	[8,13]
Multibody dynamics/gear-contact modeling	Prediction of drivetrain excitation forces, bearing reactions, and order content.	Gearbox and e-drive housing metamaterial tuning.	Provides source-informed target frequencies and loads.	Requires coupling to structural and acoustic models and validated contact parameters.	[8,10,11,12]
Transfer path analysis	Ranking of source–transfer–receiver contributions and candidate treatment locations.	Wheel arch, e-drive housing, battery tray, and body-panel treatments.	Ensures the metamaterial is placed on a dominant path.	Requires validated measurements or system models and is not a unit-cell design method.	[6,7,8,9,13]
Reduced-order/homogenized models	Efficient representation of VAMM behavior in larger system models.	Full-vehicle CAE workflows with local detailed metamaterial regions.	Reduces computational cost and supports optimization loops.	Frequency-dependent and dispersive behavior must be retained and validated.	[26,27,28,29,36,37,47,48,49,50,51,52,53]
Uncertainty and sensitivity analysis	Assessment of robustness to tolerances, material variation, interfaces, and operating conditions.	All production-relevant resonant metamaterial components.	Supports tolerancing, robust optimization, and quality-control planning.	Requires realistic parameter distributions and repeated simulations or surrogate models.	[38,42,43,44,45]

Note. The listed purposes and representative applications are grounded in the cited foundational and application studies. The stated advantages and limitations are qualitative author syntheses intended to guide method selection rather than universal rankings.

**Table 9:** Proposed order-targeted simulation workflow for EV vibroacoustic metamaterial development.

Workflow Step	Main Task	Typical Input Data	Output for the Next Step
1. NVH problem identification	Identify tonal, low-frequency, or order-related issues.	Operational measurements, order maps, subjective sound-quality feedback.	Target frequency/order and critical operating condition.
2. Source modeling	Characterize excitation mechanism.	Gear-contact multibody dynamics (MBD), electromagnetic force simulation, tire/road or compressor data.	Force spectra, order content, excitation locations.
3. Transfer path ranking	Determine dominant structural or airborne paths.	TPA, modal analysis, ODS, panel contribution, structural intensity.	Candidate component and treatment location.
4. Mechanism selection	Choose suitable VAMM mechanism.	Frequency range, available package space, material family, durability limits.	Candidate resonator, lattice, membrane, or active concept.
5. Unit-cell or local design	Tune bandgap or resonance to the target.	Material data, geometry constraints, initial analytical/lumped model.	Preliminary architecture and expected stop band.
6. Component-level simulation	Evaluate finite structure response and insertion loss.	Detailed FEM, harmonic/random response, damping and boundary data.	Predicted vibration or sound reduction and stress levels.
7. Structural–acoustic evaluation	Estimate radiated sound or cabin response.	Surface velocities, acoustic cavity or BEM model, receiver positions.	Sound power, SPL, psychoacoustic indicator changes.
8. Robustness and manufacturability check	Assess tolerance, aging, and production-route sensitivity.	Parameter scatter, temperature-dependent data, process tolerances.	Robust design window and production-intent geometry.
9. Prototype and model updating	Validate and update model using measurements.	FRF, modal test, laser Doppler vibrometry (LDV), acoustic measurements.	Validated model and revised design parameters.
10. Vehicle-level validation	Confirm benefit under realistic operation.	Road/dyno tests, cabin microphones, order tracking, durability-aged samples.	Final evidence for industrial adoption.

**Table 10:** Experimental validation methods for automotive vibroacoustic metamaterials.

Method	Validation Scale	Measured Quantity	Main Relevance for VAMMs	Key Limitation	Representative References
Impact-hammer modal testing	Unit cell, coupon, component	Natural frequencies, damping ratios, mode shapes	Checks resonator tuning and host-structure modal coupling	Limited excitation energy and local measurement coverage	[8,13,30,31]
Electrodynamic shaker testing	Coupon, component, subsystem	Controlled acceleration, velocity, FRFs, transmissibility	Allows repeatable before/after comparison and insertion-loss evaluation	Boundary conditions may differ from vehicle operation	[6,7,8,9,30,31,32,33,34]
Frequency response function measurement	Coupon to vehicle subsystem	Mobility, transfer functions, acceleration response	Directly quantifies attenuation band and detuning	Sparse sensors may miss local resonator activation	[6,7,8,9,30,31,32,33,34,38]
Scanning laser Doppler vibrometry	Coupon, panel, housing section	Full-field velocity, wave propagation, operational shapes	Visualizes bandgap behavior, wave attenuation, and local resonance	Requires optical access and careful surface preparation	[7,8,30,31]
Impedance tube testing	Acoustic material sample	Absorption coefficient, impedance, reflection, transmission	Useful for membrane, Helmholtz, and porous-meta-acoustic specimens	Simplified 1D conditions, limited structural representativeness	[23,26,27,42]
Microphone and sound power testing	Component, subsystem, vehicle	SPL, sound power, insertion loss	Connects structural changes to acoustic radiation performance	Requires controlled acoustic environment and repeatable excitation	[7,8,13,43]
Operational deflection shape analysis	Subsystem and vehicle	Operating vibration patterns and dominant radiating regions	Identifies realistic hotspots for metamaterial placement	Does not directly separate modes without additional processing	[6,7,10,11,12,13]
Durability and environmental testing	Component and vehicle subsystem	Frequency shift, fatigue damage, post-aging FRFs	Assesses whether attenuation survives automotive lifetime conditions	Long tests and limited published metamaterial-specific protocols	[38,39,44,45,46]

**Table 11:** Proposed validation roadmap for electric vehicle vibroacoustic metamaterials.

Validation Phase	Main Objective	Typical Tests	Acceptance Focus	Output for the Design Workflow
Material and resonator characterization	Identify stiffness, damping, density, resonance frequency, and manufacturing scatter	Impulse excitation, dynamic mechanical analysis (DMA) where available, modal testing, dimensional inspection	Agreement between manufactured geometry and target resonance	Material cards, resonator tuning data, uncertainty ranges
Unit-cell and coupon validation	Confirm local-resonance or bandgap mechanism under controlled conditions	FRF measurement, SLDV, shaker excitation, impedance tube for acoustic units	Measured attenuation band matches predicted band location and bandwidth	Validated unit-cell model and corrected simulation parameters
Component-level validation	Assess performance in real geometry and boundary conditions	Housing, wheel-arch, door, cover, or battery-tray tests with representative excitation	Insertion loss in structural response and reduced radiating velocity	Component-level design decision and placement optimization
Acoustic and psychoacoustic validation	Connect structural attenuation to perceived noise benefit	Microphone measurements, sound power, source localization, order tracking, psychoacoustic metrics	Reduction of target tonal component or perceptual metric, not only local vibration	Evidence of cabin-relevant NVH benefit
Environmental and fatigue validation	Verify stability over automotive operating conditions	Thermal cycling, humidity, vibration fatigue, shock, corrosion or salt exposure	Bandgap frequency, attenuation depth, and attachment integrity remain acceptable	Durability-corrected design margins and production risk assessment
Vehicle-level validation	Demonstrate effectiveness in the complete source–transfer–receiver chain	Road test, dynamometer, operating deflection shapes, operational TPA, speed sweeps	Target order or resonance is reduced at receiver level under realistic operation	Vehicle-ready evidence and final integration decision

**Table 12:** Artificial intelligence methods for vibroacoustic metamaterial design and their EV NVH relevance.

AI/Optimization Method	Primary Role	Typical Input Data	Main Output	EV NVH Relevance	Key References
Gaussian Process Regression/Kriging	Probabilistic surrogate modeling and uncertainty-aware optimization	FE bandgap datasets, geometric parameters, material properties	Predicted resonance frequency, bandgap location, uncertainty bounds	Useful when few expensive simulations are available and design robustness is important	[47,48,51]
Support Vector Regression	Nonlinear regression for limited training sets	Unit-cell dimensions, resonator parameters, damping descriptors	Bandgap frequency or attenuation prediction	Suitable for early-stage parametric studies with moderate data volume	[47,49]
Random Forest/Extra Trees	Fast prediction and feature-importance estimation	Simulation results, manufacturing variables, placement descriptors	Performance prediction and sensitivity ranking	Can identify which geometric or material variables dominate detuning risk	[47,51]
Artificial neural networks	High-dimensional nonlinear mapping	Large simulation datasets, image/topology descriptors, lattice parameters	Dispersion curves, transmission loss, insertion loss	Useful for complex unit cells and multi-resonant EV structures	[48,49,50,51]
Autoencoders and latent-space models	Dimensionality reduction and generative representation	Voxelized geometries, unit-cell images, topology descriptors	Compact design variables and candidate structures	Supports inverse design of non-intuitive lattice or resonator architectures	[50,51]
Genetic algorithms/Non-dominated Sorting Genetic Algorithm II (NSGA-II)	Global and multi-objective optimization	Discrete resonator placement, dimensions, mass and stiffness constraints	Pareto-optimal layouts and trade-off curves	Useful for minimizing added mass while targeting selected orders or radiating panels	[33,36,37]
Reinforcement learning	Sequential design and adaptive search	Design-state representation, reward based on attenuation and constraints	Stepwise optimized architecture or tuning policy	Potential future route for adaptive or active EV meta-structures	[48,50,52]
Digital twins and model updating	Lifecycle learning and performance monitoring	Simulation models, measured FRFs, operating data, manufacturing inspection	Updated performance prediction and durability assessment	Supports robust design and production-quality tracking of resonant components	[37,46,48]

**Table 13:** Data sources and target variables for EV-specific AI-assisted metamaterial development.

Data Source	Example Input Variables	Potential Target Variables	Relevant Component	Design Value
Unit-cell and dispersion simulations	Geometry parameters, material stiffness, damping, lattice spacing	Bandgap boundaries, resonance frequency, attenuation depth	Generic resonator, panel insert, lattice core	Fast screening of candidate metamaterial architectures
Component-level FEM and FEM-BEM	Housing geometry, boundary conditions, resonator placement, structural velocities	Insertion loss, wall velocity, radiated sound power	Gearbox housing, e-drive cover, door, wheel arch	Links local design choices to acoustic radiation
Multibody drivetrain models	Transmission error, gear mesh stiffness, shaft speed, bearing forces	Critical order frequencies and force amplitudes	Gearbox/e-drive housing	Defines the target frequency bands for order-tuned design
Electromagnetic motor simulations	Slot/pole combination, torque ripple, radial force harmonics, inverter switching data	Motor-order maps and force spectra	Motor casing, e-drive cover, mounts	Connects metamaterial tuning to electromagnetic excitation sources
Operational NVH measurements	Order spectra, FRFs, ODS maps, microphone signals, acceleration data	Cabin SPL, tonal prominence, psychoacoustic metrics	Full vehicle or subsystem	Validates whether local attenuation affects passenger perception
Manufacturing and quality data	Dimensional scatter, material batches, adhesive thickness, AM surface condition	Frequency shift, attenuation scatter, failure probability	All resonant metamaterial components	Supports robust design against production variability
Durability and aging data	Temperature cycles, fatigue load history, humidity, salt exposure	Post-aging resonance frequency, residual insertion loss, damage state	Wheel arch, battery casing, covers, bonded panels	Enables lifetime performance prediction and digital twin updating

**Table 14:** Sustainability opportunities and risks of vibroacoustic metamaterials for electric vehicles.

Sustainability Dimension	Potential Benefit	Main Risk	Design Implication	Relevant References
Mass reduction	Targeted attenuation can reduce the need for heavy damping sheets, barriers, or large dynamic absorbers.	Added resonators may still increase mass if coverage is not optimized.	Use insertion-loss-per-kilogram and order-specific acoustic benefit as design metrics.	[6,7,8,9,30,31,32,33,37]
Material efficiency	Geometry-driven performance can provide high acoustic benefit with limited material volume.	Complex AM parts may require supports, post-processing, or scrap if quality is poor.	Compare near-net-shape AM with scalable molding, casting, or forming routes.	[5,52,64,65,66,67,68]
Multifunctionality	One component can combine structural, acoustic, vibration, and protective roles.	Overloading one component with many functions may compromise durability or safety.	Use multi-objective optimization and keep safety-critical functions constrained.	[34,39,67]
Durability	A robust meta-structure can maintain acoustic performance without replacement.	Resonators may suffer fatigue, creep, adhesive degradation, or detuning.	Include thermal cycling, humidity, fatigue, and post-aging acoustic validation.	[38,44,45,46]
Recyclability	Monomaterial polymer or metallic designs can simplify end-of-life processing.	Hybrid resonator–damper–adhesive assemblies may be difficult to separate.	Prefer reversible joining or compatible materials where possible.	[15,52,67,68]
Manufacturing energy	Lightweight optimized structures can reduce use-phase energy demand.	Energy-intensive metal AM may offset some material-saving benefits.	Perform process-specific lifecycle comparison instead of assuming AM is greener.	[5,52,64,65,66,67,68]
Acoustic comfort and health	Lower tonal noise and improved sound quality can increase user comfort.	A dB-focused design may miss perceived tonality or annoyance.	Include psychoacoustic and order-based metrics in sustainability-oriented NVH assessment.	[1,2,3,4,40,41]
Lifecycle assessment	A functional comparison can reveal trade-offs across manufacturing, vehicle use, service, and end-of-life stages.	Results can be misleading if the functional unit, system boundary, lifetime, electricity mix, or replacement assumptions differ.	Compare equal NVH function over a stated service life and document inventory assumptions and sensitivity according to ISO 14040/14044.	[97,98,99]

**Table 15:** Suggested lifecycle-oriented assessment metrics for automotive vibroacoustic metamaterials.

Metric Category	Suggested Metric	Purpose	Why It Matters for EV NVH
Acoustic efficiency	Insertion loss per kilogram; tonal prominence reduction per kilogram	Compares acoustic benefit against added mass.	Directly evaluates whether the metamaterial outperforms conventional damping or tuned mass damper (TMD) solutions.
Order-targeted performance	Stop-band overlap with critical motor, gear, tire, or compressor orders	Checks whether the bandgap addresses a real vehicle excitation.	Prevents generic bandgaps that do not reduce cabin-relevant noise.
Structural impact	Change in stiffness, modal frequencies, fatigue safety factor, crash-relevant properties	Assesses whether NVH improvement compromises mechanical function.	Essential for e-drive housings, battery enclosures, and structural panels.
Manufacturing impact	Cycle time, scrap rate, process energy, dimensional scatter	Evaluates industrial feasibility and environmental burden.	A high-performance design must be scalable and repeatable.
Durability retention	Post-aging resonance shift and residual insertion loss	Measures lifetime NVH stability.	A detuned resonator may lose its EV-order attenuation function.
Circularity	Material compatibility, separability, recycled content, repairability	Supports end-of-life and resource-efficiency evaluation.	Multi-material acoustic packages may become recycling barriers.
System-level value	Cabin SPL, psychoacoustic metric change, component count reduction	Links local material design to vehicle-level customer perception.	The final target is perceived acoustic comfort, not only unit-cell bandgap width.
Functional equivalence	Specified dB/order reduction over a defined component service life	Ensures that the metamaterial and conventional baseline deliver the same NVH function.	Prevents misleading comparisons based only on kilograms of material or beginning-of-life peak attenuation.
Lifecycle impact	kg CO_2_-eq, primary energy demand, recycled content, recyclability/recoverability, and replacement rate	Quantifies environmental burdens and circularity across the selected system boundary.	Shows whether use-phase mass savings and component consolidation offset production and end-of-life burdens.

**Table 16:** Technology-readiness-oriented assessment of main EV vibroacoustic metamaterial application areas.

Application Area	Current Evidence Base	Approximate Readiness Interpretation	Main Industrial Barrier	Most Realistic Next Validation Step	Key References
Rear wheel arches/tire cavity path	Vehicle-oriented experimental evidence for resonant metamaterial mitigation of low-frequency tire-related structure-borne noise.	Medium–high research maturity; among the closest to near-term application.	Cost-effective manufacturing route and robust attachment in dirty, wet, and mechanically exposed locations.	Durability-tested wheel-arch demonstrator with production-representative material and mounting concept.	[7]
Gearbox/e-drive housing	Numerical and experimental evidence for locally resonant lightweight gearbox-housing concepts.	Medium maturity; high value but structurally demanding.	Preserving stiffness, bearing alignment, thermal behavior, casting feasibility, and fatigue safety.	Full e-drive housing prototype with order-based excitation, sound-power measurement, and thermal/fatigue screening.	[8]
Vehicle doors, body panels, dash structures	Component and demonstrator studies for panel-attached or integrated resonator arrays.	Medium maturity for non-critical panels; lower for structural panels.	Attachment reliability, local geometry dependence, crash-package compatibility, and detuning risk.	Curved production-like panel test with shaker, LDV, acoustic radiation, and environmental aging.	[9,30,31,32]
Power-electronics covers and auxiliary enclosures	Component-level evidence for cover-integrated VAMM concepts and tonal vibration reduction.	Medium maturity where covers are replaceable and non-primary structural parts.	Thermal exposure, electromagnetic compatibility, sealing, and serviceability.	Cover-level test under realistic thermal and operational excitation with acoustic radiation measurement.	[31]
Battery pack casings	Mostly numerical evidence for lattice and auxetic vibration mitigation.	Low–medium maturity; strong long-term potential.	Crash, thermal, sealing, fire-safety, repair, certification, and lifecycle validation.	Subscale battery-tray prototype with random vibration, thermal cycling, and structural safety assessment.	[34,35]
Active/tunable metamaterials	Laboratory and conceptual evidence for adaptive, piezoelectric, or digitally controlled systems.	Low–medium maturity for automotive production; promising for variable orders.	Sensor reliability, controller robustness, power consumption, fail-safe behavior, and cost.	Hardware-in-the-loop demonstrator tracking a swept EV order under realistic temperature and vibration.	[36,37,47,48,49,50,51,52,53]
Topological and advanced waveguiding systems	Strong theoretical and laboratory evidence in wave-control literature, limited automotive validation.	Low automotive maturity.	Complex geometry, integration into irregular vehicle structures, and lack of vehicle-level proof.	Automotive panel or beam demonstrator showing robust waveguiding under geometric imperfections.	[26,27,28,29,52,56,57,58,59,72]

**Table 17:** Proposed industrial adoption roadmap for EV vibroacoustic metamaterials.

Time Horizon	Most Realistic Components	Likely Manufacturing Route	Required Validation Evidence	Expected Value Proposition
Short term	Wheel-arch inserts, power-electronics covers, interior trim, local panel patches, auxiliary system covers.	Polymer molding, thermoforming, stamped metal inserts, limited AM for prototypes or premium applications.	Component FRF, LDV, acoustic measurement, vehicle-level A/B test, basic environmental screening.	Targeted tonal noise reduction with limited redesign and lower mass than conventional dynamic absorbers.
Mid term	E-drive housings, gearbox covers, door and dash structures, production-integrated resonator arrays.	Die casting, stamping, injection molding, hybrid manufacturing, production-representative adhesive or mechanical joining.	Order-based excitation tests, sound-power radiation, durability, thermal cycling, dimensional scatter assessment.	Integrated lightweight NVH performance designed into the component rather than added as a late corrective measure.
Long term	Battery trays, structural meta-panels, adaptive/tunable EV metamaterial systems, digital twin-monitored components.	Advanced casting, scalable lattice manufacturing, multi-material hybrid processes, embedded sensing and adaptive control.	Full vehicle validation, crash and safety certification, lifecycle assessment, digital twin updating, aging-performance tracking.	Multifunctional structures combining NVH, stiffness, crash, thermal, and lifecycle benefits.

**Table 18:** Main research gaps and recommended actions for EV vibroacoustic metamaterial development.

Research Gap	Current Limitation	EV Consequence	Priority	Recommended Action	Key References
Vehicle-level validation	Many studies remain at unit-cell, beam or plate level.	Unclear transferability to curved, damped and assembled EV structures.	High	Use component and full-vehicle A/B tests with cabin response metrics.	[6,7,8,9,30,31,32,33,34,35]
Order-targeted design	Bandgaps are often tuned to fixed frequencies.	Speed-dependent gear and motor orders may move outside the stop band.	High	Derive target bands from order maps, TPA and operating deflection shapes.	[1,6,7,8,10,11,12,13]
Durability and aging	Few studies report end-of-life acoustic performance.	Resonance detuning, fatigue or debonding may occur during service.	High	Include thermal cycling, random vibration, humidity, fatigue and post-aging NVH tests.	[38,39,44,45,46]
Manufacturing scalability	Many demonstrators rely on additive manufacturing.	Production cost and dimensional scatter may prevent industrial adoption.	High	Redesign promising concepts for molding, stamping, casting or hybrid manufacturing.	[7,8,33,34]
Benchmarking	Reported metrics vary widely across publications.	Difficult comparison with conventional NVH countermeasures.	Medium	Report insertion loss, added mass, frequency range, baseline and validation level.	[40,41,42,43,44]
AI reliability	Many ML models are trained mostly on simulation data.	Predicted designs may not survive manufacturing and validation constraints.	Medium	Use physics-informed, uncertainty-aware and experimentally updated models.	[36,37,47,48,49,50,51,52,53]
Lifecycle sustainability	Lightweight benefits are often claimed but rarely quantified.	Potentially high manufacturing energy or poor recyclability may offset benefits.	Medium	Use mass-normalized attenuation and lifecycle-oriented assessment metrics.	[39,55,64,65,66,67,68]

**Table 19:** Priority future research directions for electric vehicle vibroacoustic metamaterials.

Future Direction	Target Component	Main Objective	Required Methods	Expected Maturity Path	Main Risk
Order-targeted e-drive housing	Gearbox and motor housing	Reduce gear-whine and motor-order radiation.	MBD, EM simulation, FEM-BEM, TPA, order analysis.	Component bench → e-drive rig → vehicle A/B test.	Stiffness, bearing alignment and thermal constraints.
Meta-core battery enclosure	Battery tray and cover	Reduce road-induced vibration while preserving structural safety.	Random vibration FEM, crash analysis, thermal and durability testing.	Numerical concept → subcomponent → safety-compatible prototype.	Crashworthiness and certification complexity.
Wheel-arch and liner metamaterials	Rear/front wheel arch, liner	Mitigate tire cavity and road-noise transfer.	Vehicle dyno test, acoustic measurement, structural intensity.	Near-term passive production candidate.	Cost and attachment durability.
Trim and cover-integrated resonators	Power-electronics covers, interior trim, dash panels	Suppress local tonal panel radiation.	Modal testing, FRF, LDV, acoustic A/B test.	Low-risk component implementation.	Rattle, aging and packaging.
AI-assisted inverse design	All component classes	Shorten design loop and target known EV orders.	Surrogate models, physics-informed ML, uncertainty quantification.	Design tool integration before production use.	Black-box predictions and poor manufacturability.
Adaptive metamaterials	Premium EV e-drive and body systems	Track speed-dependent tonal orders.	Sensors, control electronics, tunable resonators, digital twins.	Laboratory → subsystem → premium low-volume vehicle.	Reliability, cost and fail-safe operation.

**Table 20:** Evidence-based engineering selection summary for EV vibroacoustic metamaterial concepts.

Concept Family	Best-Matched EV Problem/Component	Evidence and Current Readiness	Main Engineering Advantage	Main Limitation/When Not Preferred	Recommended Role and Representative References
Passive locally resonant and hybrid multi-resonant	Tire cavity paths, door and body-panel modes, covers, gearbox and e-drive housing radiation	Moderate-to-strong component evidence and selected vehicle-relevant demonstrations; highest current readiness among metamaterial families	Compact, targetable stop bands with favorable mass efficiency for stable tonal problems	Sensitive to frequency drift, attachment, damping, manufacturing scatter, and environmental aging; not a broadband solution	Preferred near-term passive option for known orders or resonances [6,7,8,9,30,31,32,33]
Lattice, auxetic, and multifunctional meta-cores	Battery trays, large enclosures, structural panels, and multifunctional supports	Promising but mainly numerical or laboratory evidence; lower automotive readiness	Can combine vibration isolation with stiffness, energy absorption, and component consolidation	Crash, thermal, sealing, mass, repairability, and host-material interactions may dominate the design	Mid-term co-design option rather than an add-on NVH treatment [34,35]
Membrane, Helmholtz, labyrinthine, and coiled-space concepts	Interior trim, compact covers, auxiliary system enclosures, and predominantly airborne paths	Strong generic acoustic evidence but limited vehicle-level structural validation	Thin packages and low-frequency acoustic tuning in restricted spaces	Narrowband response, sealing and contamination risks, temperature sensitivity, and limited load-bearing capability	Compact acoustic package or hybrid treatment when structural loading is modest [12,13,19,21]
Active and tunable meta-structures	Speed-dependent motor harmonics, moving gear orders, and operating-condition-dependent tonal problems	Mostly laboratory or concept-stage evidence	Potential to track frequency shifts and widen useful operating range	Requires sensors, electronics, power, control authority, reliability, diagnostics, and fail-safe behavior	Long-term adaptive option after passive solutions and source control are exhausted [36,37,47,48,49,50,51,52,53]
Topological and pentamode concepts	Robust waveguiding, path redirection, and advanced multifunctional structural control	Predominantly fundamental and small-scale evidence	Potential robustness to defects and unusual effective dynamic properties	Complex geometry, uncertain vehicle-scale benefit, and low manufacturing and validation maturity	Exploratory long-term research rather than a near-term production solution [17,20,24,25]
Conventional or hybrid NVH treatments	Broadband road and aerodynamic noise, uncertain excitation ranges, and low-cost mature applications	High industrial maturity and well-established validation methods	Robust, predictable, cost-effective, and broadband when mass and space permit	May conflict with lightweighting or packaging and can be inefficient at selected low-frequency tones	Mandatory baseline and often the preferred complement to a metamaterial solution [1,2,3,4,30,31]

## Data Availability

No new data were created or analyzed in this study. Data sharing is not applicable to this article.

## Abbreviations

The following abbreviations are used in this manuscript:

AI	Artificial intelligence
AM	Additive manufacturing
ASTM	ASTM International
BEM	Boundary element method
CAE	Computer-aided engineering
CAD	Computer-aided design
dB(A)	A-weighted decibel
DMA	Dynamic mechanical analysis
EM	Electromagnetic
EMC	Electromagnetic compatibility
EV	Electric vehicle
FE	Finite element
FEM	Finite element method
FEM-BEM	Coupled finite element method–boundary element method
FEM-SEA	Hybrid finite element method–statistical energy analysis
FRF	Frequency response function
HVAC	Heating, ventilation, and air conditioning
ICE	Internal combustion engine
ISO	International Organization for Standardization
LDV	Laser Doppler vibrometry
MBD	Multibody dynamics
ML	Machine learning
NSGA-II	Non-dominated Sorting Genetic Algorithm II
NVH	Noise, vibration, and harshness
ODS	Operational deflection shape
PWM	Pulse-width modulation
RMS	Root mean square
RTM	Resin transfer molding
SAE	SAE International
SEA	Statistical energy analysis
SLDV	Scanning laser Doppler vibrometry
SPL	Sound pressure level
TMD	Tuned mass damper
TPA	Transfer path analysis
TRL	Technology readiness level
UV	Ultraviolet
VAMM	Vibroacoustic metamaterial
3D	Three-dimensional
*a*	Lattice constant
*c*	Wave speed
*f*_0_	Local resonator natural frequency
*f*_Bragg_	Approximate Bragg bandgap center frequency
*k*	Effective stiffness
*m*	Resonator mass
